# Modulation of the clonal burden in patients with lower-risk myelodysplastic neoplasms treated with imetelstat

**DOI:** 10.1038/s41375-025-02831-z

**Published:** 2026-01-12

**Authors:** Valeria Santini, Amer M. Zeidan, Koen Van Eygen, Pierre Fenaux, Ulrich Germing, Yazan F. Madanat, Azra Raza, Michael R. Savona, Mikkael A. Sekeres, Sylvain Thépot, Marco G. Raddi, Thomas Boyer, Libo Sun, Ying Wan, Tymara Berry, Qi Xia, Fei Huang, Souria Dougherty, Shyamala Navada, Faye Feller, Rami S. Komrokji, Uwe Platzbecker

**Affiliations:** 1 DMSC, Hematology MDS Unit, University of Florence, AOUC, Florence, Italy; 2 Section of Hematology, Department of Internal Medicine, Yale School of Medicine and Yale Cancer Center, Yale University, New Haven, CT USA; 3 Algemeen Ziekenhuis Groeninge, Kortrijk, Belgium; 4 Saint-Louis Hospital, University of Paris 7, Paris, France; 5 Clinic for Haematology, Oncology and Clinical Immunology, Düsseldorf University Hospital, Heinrich Heine University, Düsseldorf, Germany; 6 Harold C. Simmons Comprehensive Cancer Center, University of Texas Southwestern Medical Center, Dallas, TX USA; 7 Columbia University Medical Center, New York, NY USA; 8 Vanderbilt-Ingram Cancer Center, Vanderbilt University Medical Center, Nashville, TN USA; 9 Sylvester Comprehensive Cancer Center, University of Miami, Miami, FL USA; 10 Hématologie - Maladies du Sang, Centre Hospitalo-Universitaire, Angers, France; 11 Geron Corporation, Foster City, CA USA; 12 Moffitt Cancer Center, Tampa, FL USA; 13 Department of Hematology, Cellular Therapy, Hemostaseology and Infectious Diseases, Leipzig University Hospital, Leipzig, Germany

**Keywords:** Translational research, Drug development, Drug development

## Abstract

Existing treatments for lower-risk myelodysplastic syndromes/neoplasms (LR-MDS) focus on symptom relief. Until recently, altering the disease course was rarely considered a therapeutic objective. The first-in-class, direct, competitive telomerase inhibitor, imetelstat, demonstrated significantly higher rates of red blood cell (RBC) transfusion independence (TI) versus placebo in patients with non-del(5q), RBC transfusion-dependent LR-MDS who were relapsed/refractory to or ineligible for erythropoiesis-stimulating agents in the Phase 3 IMerge study (NCT02598661). In this exploratory analysis of IMerge, patients treated with imetelstat had greater sustained reductions in variant allele frequency of multiple mutations versus placebo recipients, which was positively associated with RBC-TI duration. Subsequent analyses showed that 70% of patients with a cytogenetic response with imetelstat achieved ≥1-year RBC-TI. Additionally, higher rates of ≥1-year RBC-TI were observed in patients with maximum variant allele frequency reduction of ≥50% in *SF3B1* (58% vs. 7%), *TET2* (90% vs. 9%), *DNMT3A* (100% vs. 13%), or *ASXL1* (50% vs. 0%) and patients with ≥50% bone marrow ring sideroblast reduction (46% vs. 0%) versus patients who did not. Lastly, 60% of patients with ≥1-year RBC-TI had ≥50% reduction in telomerase activity/human telomerase reverse transcriptase RNA. These results suggest that imetelstat targets clonal progenitor cells and may modify LR-MDS biology.

## Introduction

Myelodysplastic syndromes/neoplasms (MDS) are hematopoietic stem cell cancers characterized by recurrent genetic mutations, ineffective hematopoiesis, cytopenias, morphologic bone marrow (BM) dysplasia, and propensity to transform to acute myeloid leukemia (AML) [1]. Anemia is the most frequent cytopenia in MDS, shaping the clinical presentation of the disease, and many patients require regular red blood cell (RBC) transfusions [1, 2]. Chronic transfusions provide short-term symptomatic benefit, but do not stabilize hemoglobin (Hb) levels and may induce both short- and long-term complications [3, 4]. This is most relevant in patients with lower-risk MDS (LR-MDS), according to the Revised International Prognostic Scoring System (IPSS-R), as they show a longer disease history and longer survival than patients with higher-risk MDS [2, 5, 6]. Transfusion dependence (TD) has been associated with iron overload, transfusion reactions, oscillating Hb levels with increased cardiac complications, and shorter overall survival compared with patients with transfusion-independent (TI) disease [3, 7]. Anemia in LR-MDS is routinely managed with erythropoiesis-stimulating agents (ESAs), luspatercept, lenalidomide, hypomethylating agents, and the recently approved imetelstat in efforts to maintain/achieve RBC-TI [2, 5, 8]. Although patients with RBC-TD LR-MDS may derive transient improvements in symptoms with available therapeutic options [5, 6, 9], neither ESAs nor luspatercept, the most frequently used agents, appear to exert disease-modifying activity. There is a need to identify agents with novel mechanisms that may target fundamental pathways in MDS biology.

Imetelstat is a first-in-class, direct, and competitive inhibitor of telomerase activity (TA). Preclinical studies showed imetelstat treatment inhibits TA, leading to reduced telomere length beyond a viable threshold, reduced proliferation of neoplastic hematopoietic stem and progenitor cells (HSPC), and induced apoptotic cell death of neoplastic clones [10–15]. IMerge (NCT02598661) is a multicenter, randomized, double-blind, placebo-controlled, Phase 3 study in patients with RBC-TD LR-MDS without a chromosome 5q deletion (non-del[5q]), who have relapsed, or are refractory to, or ineligible to receive ESAs [16, 17]. The Phase 3 study of IMerge met its primary endpoint, with higher ≥8-week RBC-TI rates with imetelstat versus placebo (40% vs. 15%; *p* < 0.001) [17]. Imetelstat also resulted in higher ≥24-week RBC-TI rates as the secondary endpoint compared with placebo (28% vs. 3%; *p* < 0.001) and the exploratory endpoint of ≥1-year RBC-TI rates (18% vs. 2%; *p* < 0.01) [17]. The median duration of uninterrupted RBC-TI approached 1 year in the imetelstat arm, significantly longer compared with the placebo arm (51.6 vs. 13.3 weeks). In addition, the reduction in molecular markers of MDS disease (mutation burden, cytogenetic abnormal clones) as well as in disease markers like the number of ring sideroblasts (RS) in the imetelstat arm, suggest a potential for disease modification [17].

Recurrent somatic mutations found in MDS are prognostically relevant and are involved in a variety of genetic regulation processes (e.g., splicing, epigenetic modification, DNA damage response) [1, 2]. Mutations in the spliceosome gene *SF3B1*, and epigenetic modifiers *TET2*, *DNMT3A*, and *ASXL1*, were the most frequently observed in the evaluable patient samples of the IMerge Phase 3 study [17].

Although evidence is scarce, increased TA and human telomerase reverse transcriptase (hTERT) RNA expression have been reported in MDS HSPCs [18–21]. Telomerase induction confers a growth and survival advantage to human cancer cells [22, 23], including HSPC clones giving rise to MDS [24, 25]. Negative prognostic indicators for overall survival and conversion to AML in MDS include shortened telomere length, which confers genetic instability, elevated TA, and increased hTERT expression [19, 26–32]. Therefore, targeting telomerase in MDS with imetelstat may have a disease-modifying role.

Here, we examine and report on exploratory translational outcomes associated with clinical response in patients treated with imetelstat in the IMerge study. We initially compared the impact of imetelstat and placebo on mutation variant allele frequency (VAF) and evaluated the association of VAF reduction with RBC-TI response and duration of TI [33]. These exploratory analyses led to the demonstration that the subgroup of patients who achieved sustained (≥1 year) RBC-TI with imetelstat treatment had also sustained VAF reduction over time in multiple genes [17, 34]. This publication focuses on this subgroup to further characterize mutation profile changes over time, including emerging mutations and their impact on response. The effects of imetelstat on reduction of VAF, cytogenetic clones, and RS cells in BM, along with the correlation to clinical response, was explored to elucidate the potential disease-modifying activity of imetelstat and its impact on underlying MDS disease biology. For a small subset of cases, a flow cytometric evaluation of BM erythroid maturation during treatment was also determined.

## Methods

### Study design and patient population

The full design, methodology, and results of IMerge were previously reported [17]. Patients received imetelstat sodium 7.5 mg/kg (equivalent to 7.1 mg/kg active dose) or placebo as an intravenous infusion over 2 h every 4 weeks until disease progression, unacceptable toxicity, withdrawal of consent, or lack of response.

### Ethics approval and consent to participate

The study protocol was approved by an institutional review board at each site, and the study was conducted in accordance with the International Conference on Harmonization, Good Clinical Practice guidelines, and local standard operating procedures. Written informed consent was obtained from all patients or their legal representative if they were unable to provide consent.

### Outcomes

A ≥1-year RBC-TI rate, cytogenetic response, change from baseline in mutation VAF, BM RS, TA, and hTERT RNA, were exploratory outcomes. A ≥1-year RBC -TI rate was defined as the proportion of patients without any RBC transfusion during ≥1 year starting from study day 1 until subsequent anticancer therapy, if any. Cytogenetic analysis was performed at the central laboratory by karyotyping using BM samples; cytogenetic response per International Working Group 2006 criteria [35] was assessed in those patients with abnormal baseline cytogenetics by an independent review committee. Assessment of BM RS was centralized and performed by manual absolute count after Perls’ staining.

Sequencing of 36 genes commonly associated with MDS were tested by next-generation sequencing (NGS) on peripheral blood samples taken at baseline (pretreatment) and every 12 weeks after treatment to assess mutation VAF change by treatment over time. VAF for patients with ≥10% mutational VAF at baseline and ≥1 assessment after treatment was evaluated. An emerging mutation during treatment was defined as VAF ≥10% at any time point after treatment for patients with undetectable or below 5% (the lower sensitivity threshold) VAF at baseline. After DNA extraction from leukocytes, targeted, amplicon-based NGS was performed at Quest Diagnostics using DNA bait capture methodology on the NextSeq^®^ (Illumina^®^) platform and a LeukoVantage^®^ MDS gene panel [17]. The lower sensitivity threshold was 5% mutated alleles in a mixed population, and the size of the clonal population was assessed by percentage of mutation reads reported.

Additionally, for 4 illustrative cases enrolled in the study at the MDS Unit, Hematology, DMSC University of Florence, AOUC, Florence, Italy, BM mononuclear cells were separated by density gradient centrifugation and DNA extracted at different time points included at baseline and during treatment with either imetelstat or placebo (at >6 and >12 months from the first given dose of imetelstat/placebo).

Additionally, multiparametric flow cytometry (MFC) analysis from fresh BM aspirates was performed for the same 4 patients, at baseline and during treatment with either imetelstat or placebo (at +6 and +12 months; data at +24 months were also available for the imetelstat ongoing responder). Overall, 1 × 10^6^ BM cells were collected, stained for surface markers, and then washed using FACS Lyse (BD Biosciences, San Jose, CA) to remove enucleated RBCs. The following panel of antibodies used included: CD45 V-500 (clone 2D1), HLA-DR V-450 (clone G46-6), CD36 FITC (clone CLB-IVC7), CD105 PE-CF594 (clone 266), CD34 PerCp-cy5.5 (clone 8G12), CD117 PE-cy7 (clone 104D2), CD33 APC (clone P67.6), and CD71 APC-H7 (clone L01.1), which were all purchased from BD Biosciences. A total of 150,000 events per sample were acquired by FACSCanto™ II (BD Biosciences, San Jose, CA) and data were analyzed with Infinicyt™ software (Cytognos S.L., Salamanca, Spain). The gating strategy was applied on the total BM erythroid population, which was defined as SSC^low^/CD36^+^/CD71^+^/CD45−/dim, and the immature stages of erythroid maturations were identified according to the recommendations from iMDS Flow Working Group [36]. Next, myeloid and erythroid progenitors were gated as previously described [37]. All MFC analyses were performed blinded of outcome to imetelstat/placebo.

TA was measured from peripheral blood mononuclear cells at baseline and post dose during cycles 1 and 2 using the quantitative telomere repeat amplification protocol; RNA was purified from blood and hTERT RNA expression levels were assessed by reverse transcription polymerase chain reaction [16].

### Analysis

The present analyses, with a clinical cutoff date of October 13, 2023, retrospectively evaluated associations between achieving ≥1-year RBC-TI and cytogenetic response. VAF reduction, and BM RS reduction, as well as the associations among VAF reduction, BM RS reduction, and changes in Hb using Pearson correlation and logistic regression. Summary statistics were provided for continuous and categorical variables. All analyses were performed using Statistical Analysis Software (version 9.4) and R (version 4.2.2).

## Results

A total of 178 patients (*n* = 118 imetelstat; *n* = 60 placebo) were included in the intention-to-treat population. At the cutoff date, of the 118 patients who received imetelstat in Phase 3 of IMerge, 21 (18%; 95% confidence interval, 11–36) had achieved ≥1-year RBC-TI, 26 (22%; 95% confidence interval, 15–31) patients had achieved ≥8-week but <1-year RBC-TI, and 71 (60%) had <8-week RBC-TI and were considered nonresponders. Baseline demographic and disease characteristics for patients in the imetelstat arm grouped by RBC-TI response and the placebo arm were balanced (Table 1).

**Table 1:** Baseline patient and disease characteristics.

Characteristic	Patients who achieved ≥1-year RBC-TI with imetelstat (*N* = 21)	Patients who achieved ≥8 weeks to <1-year RBC-TI with imetelstat (*N* = 26)	Imetelstat nonresponders (*N* = 71)	Placebo (*N* = 60)
Age (years), median (range)	73 (52–83)	72 (61–86)	71 (44–87)	73 (39–85)
Male sex, *n* (%)	12 (57)	18 (69)	41 (58)	40 (67)
Time since original diagnosis of MDS (years), median (range)	3.0 (0.1–10.4)	4.2 (0.3–13.1)	3.8 (0.5–26.7)	2.8 (0.2–25.7)
RS status, *n* (%)				
RS+	15 (71)	18 (69)	40 (56)	37 (62)
RS−	6 (29)	8 (31)	30 (42)	23 (38)
IPSS category, *n* (%)				
Low	14 (67)	18 (69)	48 (68)	39 (65)
Intermediate-1	7 (33)	8 (31)	23 (32)	21 (35)
IPSS-R category, *n* (%)				
Very low	0	0	3 (4)	2 (3)
Low	15 (71)	22 (85)	50 (70)	46 (77)
Intermediate	4 (19)	3 (12)	13 (18)	8 (13)
High	0	1 (4)	0	0
Very high	0	0	0	0
Missing	2 (10)	0	5 (7)	4 (7)
IPSS-M category, *n* (%)				
Very low/low	13 (62)	20 (77)	36 (51)	33 (55)
Moderate low/moderate high	3 (14)	3 (12)	23 (32)	16 (27)
High/very high	0	2 (8)	3 (4)	3 (5)
Missing	5 (24)	1 (4)	9 (13)	8 (13)
Prior RBC transfusion burden (RBC U/8 weeks), median (range)	6 (4-9)	6 (4-12)	7 (4-33)	6 (4-13)
Prior RBC transfusion burden, *n* (%)				
≤6 U	13 (62)	15 (58)	34 (48)	33 (55)
>6 U	8 (38)	11 (42)	37 (52)	27 (45)
Pretreatment Hb level (g/dL), median (range)	7.8 (6.5–8.8)	8.0 (6.8–9.2)	8.0 (5.3–10.1)	7.8 (6.1–9.2)
Median serum EPO level, *n* (%)				
≤500 mU/mL	18 (86)	21 (81)	48 (68)	36 (60)
>500 mU/mL	3 (14)	4 (15)	19 (27)	22 (37)
Missing	0	1 (4)	4 (6)	2 (3)
Prior ESA, *n* (%)	19 (91)	24 (92)	65 (92)	52 (87)
Prior luspatercept, *n* (%)	1 (5)	0	7 (10)	4 (7)
Cytogenetics, *n* (%)				
Normal karyotype	12 (57)	18 (69)	53 (75)	43 (72)
Abnormal karyotype	7 (33)	8 (31)	13 (18)	13 (22)
Missing	2 (10)	0	5 (7)	4 (7)

*EPO* erythropoietin, *ESA* erythropoiesis-stimulating agent, *Hb* hemoglobin, *IPSS* International Prognostic Scoring System, *IPSS-M* Molecular International Prognostic Scoring System, *IPSS-R* Revised International Prognostic Scoring System, *MDS* myelodysplastic syndromes, *RBC* red blood cell, *RS* ring sideroblast, *TI* transfusion independence, *U* units, *WHO* World Health Organization.

### Modulation of somatic mutation VAFs during imetelstat treatment and response

**Fig. 1:**
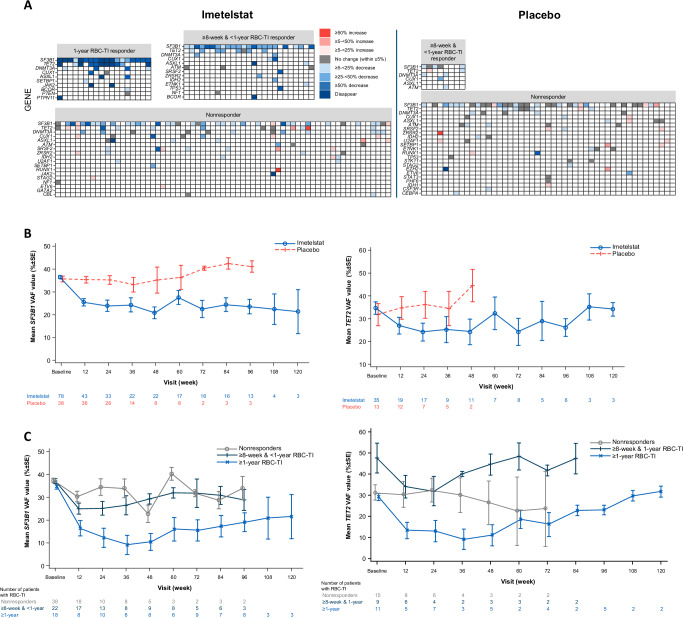
Effects on VAF reduction in imetelstat-treated patients and placebo recipients. **A** Heat map for maximum percent reduction of mutational VAF in the overall patient population.^a^ Patients rank-ordered by RBC-TI duration with the longest RBC-TI on the left and shortest RBC-TI on the right of the panel. **B** Mean value of absolute VAF in *SF3B1* and *TET2* mutations over time in the overall patient population grouped by treatment arm.^b^ **C** Mean value of absolute VAF in *SF3B1* and *TET2* mutations over time in the imetelstat treatment arm grouped by RBC-TI response.^b^ ^a^The percentage change from baseline in mutational VAF was evaluated for patients with VAF value ≥10% at baseline and ≥1 posttreatment mutation assessment. ^b^Patients who received ≥1 dose of study drug and had baseline mutation of *SF3B1* or *TET2* and ≥1 postbaseline assessment were included. Data points with only 1 ongoing patient remaining are not shown. RBC red blood cell, SE standard error, TI transfusion independence, VAF variant allele frequency.

Among 178 patients in Phase 3 of IMerge, 165 (93%) patients had baseline mutation data available, of which 98% had ≥1 MDS-related mutation. The most frequently mutated genes in this study were *SF3B1* (76% overall: 75% imetelstat group and 78% placebo group), *TET2* (33% overall: 36% and 26%, respectively), *DNMT3A* (17% overall: 17% and 16%, respectively), and *ASXL1* (15% overall: 16% and 11%, respectively). Maximum percent reductions from baseline of VAF during treatment were greater with imetelstat than with placebo in the overall patient population (Fig. 1A). In the imetelstat arm, the greater maximum percent reduction of VAF was observed predominantly in ≥1-year RBC-TI responders followed by ≥8 weeks to <1-year RBC-TI responders, whereas in the placebo arm there was minimal impact on VAF (Fig. 1A). The majority (*n* = 18/21; 86%) of patients who achieved ≥1-year RBC-TI with imetelstat had evaluable mutation data to assess VAF change from baseline. Most of the ≥1-year RBC-TI responders (*n* = 16/18; 89%) had maximum VAF reduction ≥50% with imetelstat, including 9 (*n* = 9/18; 50%) patients with complete elimination of mutations of some genes (Fig. 1A). In particular, for the most frequent *SF3B1* and *TET2* mutations, imetelstat therapy reduced VAF at week 12 (the earliest measured time point) and importantly, these reductions were sustained over time, while placebo had no impact (Fig. 1B). Furthermore, the greater reductions in mean VAF values of *SF3B1* and *TET2* during imetelstat treatment were observed in ≥1-year RBC-TI responders compared with ≥8 weeks to <1-year TI responders or to nonresponders (Fig. 1C). Absolute VAF values for mutated genes over time in individual patients showed different patterns of change in relation to dose modifications and RBC-TI events during imetelstat treatment (Supplementary Fig. 1).

**Fig. 2:**
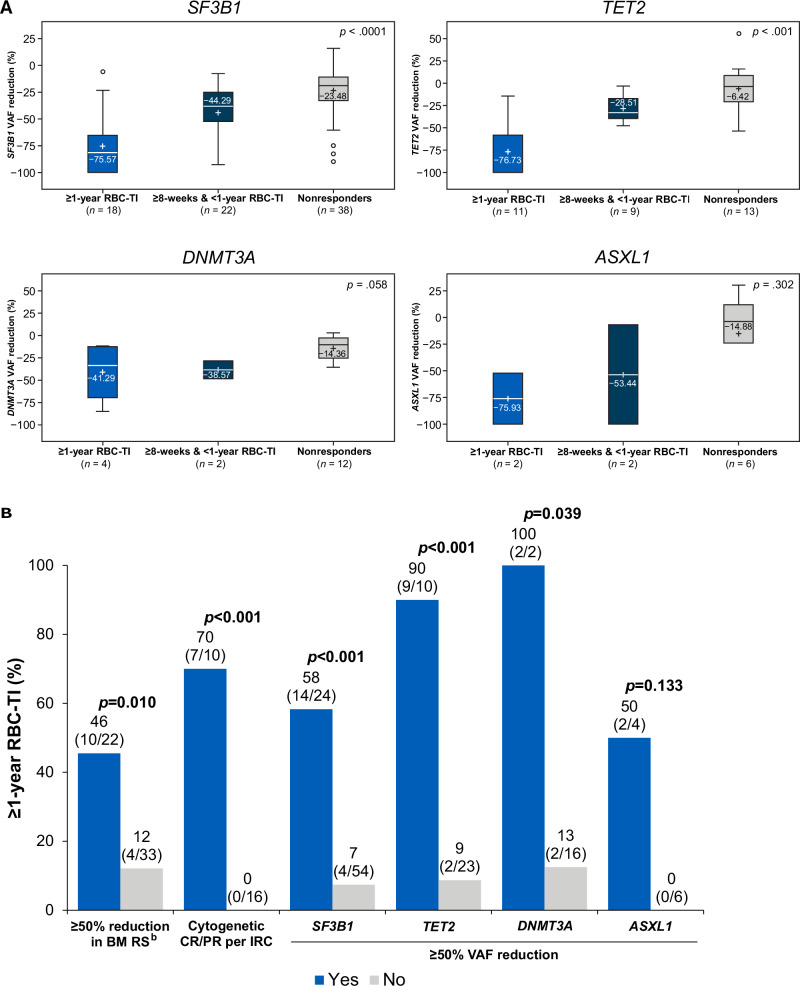
Association between VAF reduction and RBC-TI. **A** Maximum percent VAF reduction from baseline in select genes by RBC-TI response in patients treated with imetelstat.^a^ **B** ≥1-year RBC-TI rate comparison between patients with or without change in other disease modification indicators with imetelstat treatment. ^a^The *p* values are based on type III sums of squares from analysis of variance model for equality of means. Total *n*’s represent patients in the imetelstat arm who had a VAF assessment ≥10 at baseline and had ≥1 postbaseline mutation assessment. ^b^RS-negative patients were not included in the analysis of reduction in BM RS. BM bone marrow, CR complete response, IRC independent review committee, PR partial response, RBC red blood cell, RS ring sideroblast, TI transfusion independence, VAF variant allele frequency. ○ Represents an outlier observation.

Overall, patients who were treated with imetelstat and achieved ≥1-year RBC-TI had significantly greater mean values in maximum percent reduction from baseline in *SF3B1* (−76%) and *TET2* (−77%) VAF, compared with patients who achieved ≥8-week but <1-year RBC-TI response (−44% and −29%, respectively); or with nonresponders, who had the smallest VAF reduction (−24% and −6%, respectively; Fig. 2A). Similar trends were generally observed for the other key MDS-related genes, *DNMT3A* (−41% and −39%) and *ASXL1* (−76% and −53%; Fig. 2A). Logistic regression analyses suggested that as the maximum percent reduction of VAF from baseline in *SF3B1*, *TET2*, *DNMT3A*, and *ASXL1* increased by 10%, the odds of experiencing a ≥1-year RBC-TI response also increased by 67%, 112%, 62%, and 28%, respectively (Supplementary Fig. 2).

An effect with imetelstat on cytogenetic response and mutant allele burden of commonly mutated genes in MDS, indicating a reduction in abnormal clones, was previously reported in IMerge [17]. Further analysis showed that in patients who had a cytogenetic response with imetelstat, 70% achieved ≥1-year RBC-TI response, whereas of the patients who did not have a cytogenetic response, none (0%) achieved ≥1-year RBC-TI response (Fig. 2B). Similarly, higher rates of ≥1-year RBC-TI were observed in patients who had ≥50% BM RS reduction (46% vs. 0%) and patients with maximum VAF reduction of ≥50% in *SF3B1* (58% vs. 7%), *TET2* (90% vs. 9%), *DNMT3A* (100% vs. 13%), or *ASXL1* (50% vs. 0%) also had substantially higher rates of ≥1-year RBC-TI compared with those patients who did not. These data suggest that these potential disease modification indicators were associated with achievement of ≥1-year RBC-TI in patients treated with imetelstat.

**Fig. 3:**
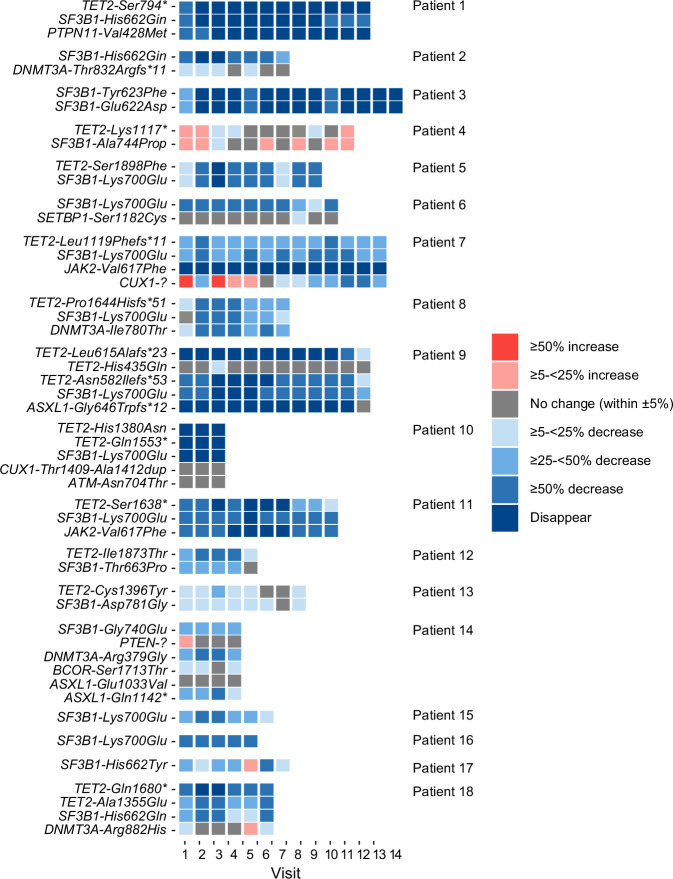
Heatmap for mutational VAF percent change over time in patients with ≥1-year RBC-TI with imetelstat.^a^ ^a^Emerging mutations were not included (see Table 2). RBC red blood cell, TI transfusion independence, VAF variant allele frequency.

Since VAFs were most substantially reduced in patients who achieved ≥1-year RBC-TI response with imetelstat, the subsequent analyses were mostly focused on the evaluable subgroup of 18 patients. The heatmap of VAF percent change from baseline over time in imetelstat-treated ≥1-year RBC-TI responders illustrated that sustained ≥50% VAF reductions over time were observed in multiple genes in 16/18 patients; 2 patients (#4 and #13) had limited VAF reduction but still achieved TI duration of 137 weeks and 84 weeks before loss of response (Fig. 3, Supplementary Table 2, and Supplementary Fig. 1). One patient (#7) exhibited an initial ≥50% increase in *CUX1* VAF followed by a gradual reduction and achieved ≥50% decrease at a later time point (VAF = 14% at baseline, increasing to 26% at posttreatment visit 1, decreasing to 6% at posttreatment visit 11), while the mutations of *TET2*, *SF3B1*, and *JAK2* had reduced VAF ≥50% with imetelstat treatment (Fig. 3 and Table 2 for absolute VAF change over time).

**Table 2:** VAF over time in 6 patients with emerging mutations but achieved ≥1-year RBC-TI with imetelstat.

Patient ID	Gene	Mutation	Baseline	Posttreatment visits												Loss of RBC-TI response at onset of emerging mutation
				1	2	3	4	5	6	7	8	9	10	11	12	
**1**	*SF3B1*	p.His662Gln	38	15	0	0	0	0	0	0	0	0	1	1	1	No
	*TET2*	p.Ser794*	40	14	0	0	0	0	0	0	0	0	0	0	0	
	*PTPN11*	p.Val428Met	35	15	3	0	0	0	0	0	0	0	0	0	0	
	***TET2***	p.Ser1688Phefs*4	***0***	***0***	***0***	***8***	***10***	***13***	***13***	***12***	***15***	***15***	***15***	***14***	***17***	
**2**	*DNMT3A*	p.Thr832Argfs*11	49	46	43	46	47	44	47	49	44					No
	*SF3B1*	p.Lys700Glu	39	12	0	0	5	9	17	25	28					
	***CBL***	***p.Cys404Phe***	***0***	***14***	***20***	***22***	***20***	***19***	***14***	***10***	***7***					
**7**	*JAK2*	p.Val617Phe	47	0	0	0	0	0	0	0	0	0	0	0	0	No
	*CUX1*	p.?	14	26	9	22	16	16	14	12	11	9	7	6	6	
	*TET2*	p.Glu184Serfs*23	7	7	4	8	9	8	12	11	19	11	10	13	22	
	*TET2*	p.Leu1119Phefs*11	77	49	32	58	54	47	51	49	48	39	38	41	45	
	*SF3B1*	p.Lys700Glu	41	24	9	23	21	20	21	19	29	16	18	19	27	
	***TET2***	***p.Asp1376Asn***	***0***	***8***	***16***	***19***	***18***	***16***	***19***	***21***	***15***	***15***	***13***	***22***	***14***	
	***CBL***	***p.Val431Ala***	***3***	***4***	***0***	***5***	***9***	***9***	***17***	***17***	***25***	***15***	***14***	***23***	***37***	
	***KRAS***	***p.Ala146Val***	***0***	***8***	***15***	***20***	***20***	***15***	***19***	***21***	***14***	***14***	***12***	***19***	***13***	
	***ASXL1***	***p.Thr600Ala***	***0***	***9***	***17***	***20***	***21***	***16***	***21***	***22***	***15***	***16***	***14***	***21***	***14***	
**9**	*ASXL1*	p.Gly646Trpfs*12	12	0	0	0	0	0	0	0	0	0	0	0	13	No
	*SF3B1*	p.Lys700Glu	37	12	6	0	0	0	3	5	5	5	6	10	26	
	*TET2*	p.Asn582Ilefs*53	33	11	5	0	0	0	0	5	4	5	5	10	25	
	*TET2*	p.His435Gln	49	51	51	46	50	48	49	48	50	50	49	49	49	
	*TET2*	p.Leu615Alafs*23	17	0	0	0	0	0	0	0	0	0	0	3	13	
	***TP53***	p.Arg196*	***0***	***0***	***0***	***0***	***0***	***0***	***0***	***0***	***0***	***0***	***0***	***6***	***22***	
**11**	*JAK2*	p.Val617Phe	36	8	4	3	0	0	0	0	15	15	17			No until other previously reduced VAFs rebound
	*TET2*	p.Ser1638*	23	5	4	0	3	0	0	0	16	16	19			
	*SF3B1*	p.Lys700Glu	41	11	5	5	3	0	3	3	17	18	19			
	***DNMT3A***	***p.Arg635Trp***	***0***	***14***	***21***	***20***	***25***	***28***	***29***	***29***	***22***	***18***	***17***			
**12**	*TET2*	p.Ile1873Thr	56	30	22	26	28	45								No
	*SF3B1*	p.Thr663Pro	30	17	18	21	18	31								
	***TET2***	p.Thr425Leufs*2	***0***	***0***	***0***	***5***	***6***	***12***								

Emerging mutation (indicated in** bold** and *italic*) during the treatment was defined as VAF ≥10% at any posttreatment time point for patients with undetectable or below 5% (the lower sensitivity threshold) VAF at baseline. *RBC* red blood cell, *TI* transfusion independence, *VAF* variant allele frequency.

### Clonal evolution and emerging mutations while receiving imetelstat: their association with RBC-TI response in patients with ≥1-year TI

Six patients developed new emerging mutations with maximum VAF reached in *TET2* (22%), *CBL* (37%), *KRAS* (21%), *ASXL1* (22%), *DNMT3A* (29%), or *TP53* (22%) genes during treatment, although VAF reductions in other pre-existing baseline mutations were observed in all cases, with complete eradication in 5 of the 6 patients (Table 2). The acquired mutations in *TET2*, *CBL*, *KRAS*, *ASXL1*, and *DNMT3A* genes were early events, detected at week 12 posttreatment, with absolute VAF values gradually increasing to the maximum VAF (range, 21–37%) over time, while the acquired mutation in the *TP53* gene emerged later, with a VAF of 6% after treatment for 96 weeks, increasing to VAF of 22% by week 108. This patient (#9) did not lose RBC-TI response.

Importantly, the onset of these emerging mutations did not lead to loss of RBC-TI response, with a range of continued follow-up of ~14–142 weeks after additional mutation onset (Supplementary Fig. 1). Among the 6 patients with emerging mutations, 5 maintained their RBC-TI response, including patient #7 who had increased *CUX1* VAF and also acquired mutations in four genes (*TET2*, *CBL*, *KRAS*, *ASXL1*) while on treatment. Patient #9 developed a *TP53* mutation and continued to respond after the mutation was detected, remaining on treatment with no progression at the time of this data cut (Table 2 and Supplementary Fig. 1). Patient #11 maintained 94 weeks of RBC-TI response in the presence of an emerging mutation in *DNMT3A* from week 12, with VAF increasing from 14% to 29% over time and loss of RBC-TI response only after all three previously eliminated mutations in *JAK2*, *TET2*, and *SF3B1* genes re-emerged to detectable VAF levels of 15–17% (Table 2 and Supplementary Fig. 1). This patient discontinued treatment due to becoming RBC-TD.

### Characteristics of patients who achieved ≥1-year RBC-TI response with imetelstat treatment

**Fig. 4:**
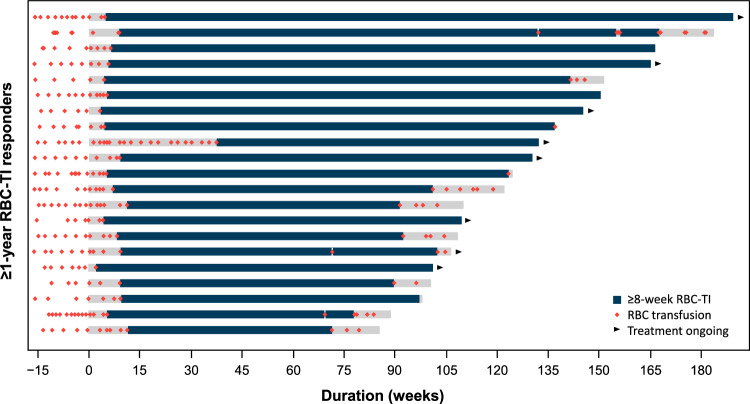
Transfusion history and RBC-TI intervals for patients who achieved ≥1-year RBC-TI with imetelstat treatment. RBC red blood cell, TI transfusion independence.

Rate of ≥1-year RBC-TI was similar in patients regardless of sex, age, time since initial diagnosis, World Health Organization (WHO) classification status, prior ESA use, Eastern Cooperative Oncology Group performance status, and IPSS or IPSS-R score risk category (Supplementary Table 1). All imetelstat-treated patients who achieved ≥1-year RBC-TI had baseline high transfusion burden; their transfusion history, including pretreatment transfusion burden, is presented in Fig. 4. Median treatment duration in patients with ≥1-year uninterrupted RBC-TI was 26.2 months, and median increase in Hb during the longest RBC-TI interval was 5.18 g/dL (interquartile range, 4.17–6.53).

### Cytogenetic response in ≥1-year RBC-TI responders

Central lab karyotyping data were available for 19/21 patients (90%) who achieved ≥1-year RBC-TI with imetelstat; 7/21 (33%) of these patients had an abnormal karyotype at baseline and were evaluable for cytogenetic response, as assessed by an independent review committee. All 7 patients achieved a cytogenetic response. Complete responses (*n* = 5/7) were recorded in patients with +8 (*n* = 2/5), with +8/−Y (*n* = 1/5), with del(13)(q12q14) (*n* = 1/5), and with +21 (*n* = 1/5). Partial responses (*n* = 2/7) were achieved in 1 patient each with +8/del(13)(q12q14) and −Y. Among the patients who achieved a cytogenetic response, 5 had mutation data available, with 4/5 (80%) showing 100% reduction in VAF and 1 85% VAF reduction (Supplementary Table 2).

### BM RS reduction in patients who achieved ≥1-year RBC-TI with imetelstat and correlation with* SF3B1* VAF and Hb levels

**Fig. 5:**
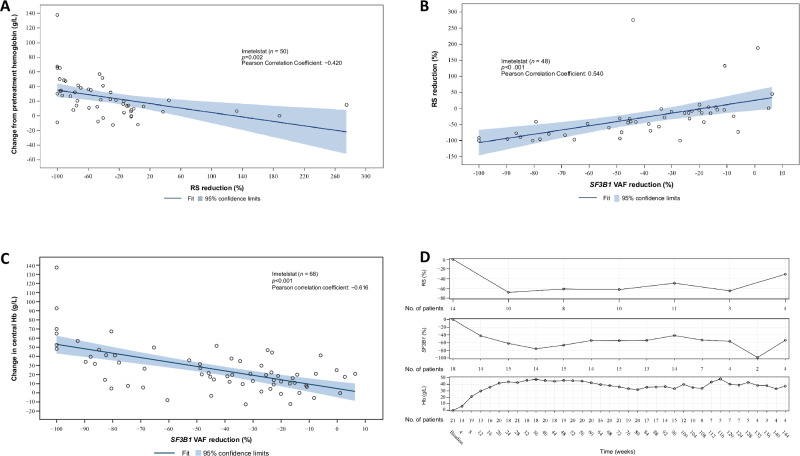
Correlation between reductions in *SF3B1* VAF, BM RS cells, and central Hb increase in the cohort of patients treated with imetelstat. **A** Correlation between maximum change in Hb from baseline and maximum percentage reduction from baseline in BM RS in patients treated with imetelstat.^a^ **B** Correlation between maximum percentage reduction from baseline in BM RS and maximum percentage reduction from baseline in *SF3B1* VAF in patients treated with imetelstat.^b^ **C** Correlation between maximum VAF percentage reduction in *SF3B1* versus maximum Hb change from baseline in the population with both VAF and central Hb data available (*N* = 68). **D** Mean VAF percentage reduction from baseline in *SF3B1*, mean change in central Hb levels from the minimum Hb level in patients who achieved ≥1-year RBC-TI, and mean percentage change from baseline in BM RS cells in RS-positive patients with MDS who achieved ≥1-year RBC-TI. ^a^The fitted line and *p* value are based on linear regression with maximum change in central Hb from pretreatment Hb as dependent variable and the maximum percentage reduction from baseline in central BM RS as independent variable. *N* represents imetelstat-treated patients who were RS positive and had baseline RS ≥15% with ≥1 postbaseline assessment. ^b^The fitted line and *p* value are based on linear regression with maximum reduction in central BM RS as dependent variable and the maximum percentage reduction from baseline in *SF3B1* VAF as independent variable. *N* represents imetelstat-treated patients who were RS positive, had baseline assessment ≥10% in *SF3B1* and had baseline RS ≥15% with ≥1 postbaseline assessment. BM bone marrow, Hb hemoglobin, RBC red blood cell, RS ring sideroblast, TI transfusion independence, VAF variant allele frequency.

Among all patients treated with imetelstat, the maximum percentage reduction in RS cells significantly correlated with maximum Hb increase from baseline (Pearson correlation coefficient = −0.420; *p* = 0.002), and maximum *SF3B1* VAF reduction from baseline (Pearson correlation coefficient = 0.540; *p* < 0.001) (Fig. 5A, B); and the maximum percentage *SF3B1* VAF reduction was correlated with increases in Hb levels (Pearson correlation coefficient = −0.616; *p* < 0.001; Fig. 5C). Furthermore, in patients with ≥1-year RBC-TI, the trend in mean values of percentage reduction by imetelstat over time in RS cells, *SF3B1* VAF reduction, and increases in Hb levels were well tracked (Fig. 5D).

Fifteen of ≥1-year RBC-TI responders (*n* = 21) were classified as RS-positive MDS and 14 had BM RS data available for central assessment; the median percentage of RS cells at baseline was 89% (33–96%). All 14 evaluable patients had a maximum percentage reduction of >30% in RS burden, with 10 (71%) achieving a ≥50% reduction of RS cells, including 3 (21%) with complete elimination of RS cells from BM (Supplementary Table 2).

### Erythroid maturation and VAF change in BM cells associated with RBC-TI response

As additional evidence, identification of somatic mutations by NGS and complete erythroid maturation characterization with MFC were performed on BM mononuclear cells collected from 4 patients treated at a single institution. These patients were selected based on complete available data on BM NGS and cytometry at baseline and specific time points, and with different treatment outcomes as follows: they were 3 imetelstat-treated patients, 1 ≥1-year RBC-TI ongoing responder, 1 ≥8 weeks to <1-year RBC-TI responder, and 1 nonresponder, along with 1 placebo patient, >12 months, with an additional 24 months’ evaluation for the imetelstat ≥1-year RBC-TI ongoing responder. Baseline characteristics for these patients are presented in Supplementary Table 3. Normalization of erythroid maturation parameter (i.e., increase in the ratio between late and early erythroid cells) and decrease in total erythroid cells (erythroid expansion considered as a marker of ineffective erythropoiesis) with concomitant reduction of VAFs in *SF3B1* and *TET2* mutations in BM samples were observed in the imetelstat RBC-TI responder but not in the imetelstat nonresponder or placebo recipient (Supplementary Fig. 3). The patient who initially responded with RBC-TI (at 6 months) and subsequently relapsed (≥8 weeks to <1-year RBC-TI responder), showed an initial decrease of VAF in *SF3B1* and *TET2* mutations during the RBC-TI response, paralleled by signs of improved erythroid maturation. Subsequently, the patient’s VAF of the mutated genes rebounded, and was accompanied by a reduction of the erythroid ratio at the time of loss of RBC-TI (at 12 months). In contrast, the ≥1-year RBC-TI ongoing responder had sustained decrease in VAF over time and an increase in erythroid precursor ratio from 2.4 at baseline to 3.5 at 12 months, and to 7.3 at 24 months with imetelstat treatment as well as a decrease in total erythroid cells from 29% to 16% and to 11%, respectively, indicating normalization of erythroid maturation indices. This correlated clinically with a maximum Hb increase of 55.5 g/L during the transfusion-free interval.

### TA/hTERT RNA reduction in patients who achieved ≥1-year RBC-TI with imetelstat

Data to evaluate the change of TA/hTERT RNA from baseline was available for 20 (95%) patients with ≥1-year RBC-TI with imetelstat. Seventeen patients (85%) had a TA/hTERT RNA maximum percentage reduction from baseline ranging from −35% to −95%, with a mean maximum percentage reduction of 65% (Supplementary Table 2). TA/hTERT RNA maximum reduction of ≥50% was achieved in 12 (60%) patients, and 5 (25%) achieved a maximum reduction of ≥30% to <50%. Furthermore, 12/20 (60%) had a baseline hTERT RNA level that exceeded the median hTERT RNA level of the overall study population. Among these patients with mutations (*SF3B1*, *TET2*, *DNMT3A*, *ASXL1*, *CUX1*, *JAK2*, *ATM*, *BCOR*, *PTEN, PTPN11, SETBP1*) and TA/hTERT data, VAF reductions ranged from 14% to 100% and TA/hTERT reductions ranged from 43% to 94% (Supplementary Table 2).

## Discussion

In this analysis of IMerge, patients treated with imetelstat had greater sustained maximum reductions in the VAFs of multiple mutations compared with patients who received placebo. The magnitude of reductions in VAF by imetelstat was positively associated with duration of RBC-TI, and VAF reduction was more substantial in patients with ≥1-year RBC-TI. This observation led to further focused analyses in this subgroup of patients with ≥1-year RBC-TI, which showed that the relevant clinical benefit of imetelstat was accompanied by significantly increased Hb levels, as expected, and by the following observations. First, in patients with ≥1-year RBC-TI response, cytogenetic response was seen in all patients with abnormal karyotype at baseline, suggesting the cytogenetic response was associated with ≥1-year RBC-TI. Second, there was substantial reductions in VAF of somatic mutations in multiple genes, including *SF3B1*, that correlated with increased Hb levels, indicating that longer RBC-TI response is associated with deeper VAF reduction. Third, data from BM aspirates confirmed the association between VAF reduction, improved erythroid maturation and RBC-TI response to imetelstat. Fourth, a reduction in BM RS cells correlated with reduction of *SF3B1* VAF and an increase of Hb levels by imetelstat treatment. Lastly, reduced TA/hTERT RNA expression was observed, demonstrating the on-target activity of imetelstat in patients with ≥1-year RBC-TI response. This finding is consistent with the IMerge Phase 2 results, where a ≥50% reduction in TA/hTERT RNA was significantly associated with higher rates of ≥8-week and ≥24-week RBC-TI [16]. Altogether, the data support the disease-modifying potential of imetelstat.

The *SF3B1* gene is mutated in ~80% of MDS with RS, and it is significantly associated with lower Hb values with respect to other MDS subtypes, which is consistent with a high degree of ineffective erythropoiesis resulting in severe anemia [38]. Most imetelstat-treated patients with ≥1-year RBC-TI had sustained VAF reduction of *SF3B1* over time, which correlated with an increase of Hb levels, leading to longer uninterrupted RBC-TI responses. These results suggest that the reduction/elimination of *SF3B1*-mutated clones by imetelstat was associated with improved erythropoiesis, in increased Hb levels and in durable RBC-TI, potentially driven by telomerase inhibition. These findings are further supported by the lack of effect on *SF3B1* VAF in placebo-treated patients.

In ≥1-year RBC-TI responders treated with imetelstat for a median duration of 26.2 months, clonal evolution analysis revealed emerging mutations in *TET2*, *CBL*, *KRAS*, *ASXL1*, *DNMT3A*, or *TP53* genes in 6 patients. It has previously been reported that *TET2* mutations can lead to aberrant DNA methylation, which is significantly associated with accelerated progression to AML [39]. Although acquired mutations in *TET2* were observed in 3 patients with ≥1-year RBC-TI response with imetelstat, this was also observed in 1 patient who received placebo with a gradual VAF increase to 41% (data not shown); however, in the imetelstat-treated case, the acquired mutation in *TET2* was not accompanied by loss of RBC-TI, suggesting that emerging *TET2* mutations do not influence the course of disease. At the time of this data cut, 2 of the 3 patients with *TET2*-acquired mutations were still responding to treatment, and 1 patient maintained RBC-TI response but came off study treatment due to an adverse event. The acquisition and/or expansion of *CBL* mutant clones is frequently observed during secondary AML transformation in high-risk patients with MDS [40]. In the present analysis, acquired mutations in *CBL* genes were observed in 2 patients, with no subsequent loss of RBC-TI response or disease progression. One ≥1-year RBC-TI responder had acquired mutations in the *TP53* gene without progression to AML after ~14 weeks of follow-up after emergence of the mutation. Importantly, the onset of these emerging mutations did not lead to loss of RBC-TI response in any of the 6 patients, even in the patient with acquired co-mutations in four genes and in the 1 patient with an acquired mutation in the *TP53* gene. Further, 1 patient maintained RBC-TI response for 93 weeks even in the presence of an emerging mutation in *DNMT3A* and ultimately lost RBC-TI response only after all three previously eliminated VAFs of *JAK2, TET2*, and *SF3B1* genes rebounded to 15–17%. This suggests that the emerging mutation in *DNMT3A* with VAF of 29% did not impact imetelstat response. Loss of RBC-TI response in this patient was proceeded by re-emergence of previously suppressed mutation clones.

Second-line treatment after ESA failure/ineligibility constitutes an unmet need for the majority of patients with LR-MDS. ESAs have not been shown to exert any disease-modifying activity [41]. Luspatercept is approved for the treatment of anemia in certain RBC-TD patients with LR-MDS, and as a transforming growth factor-β family ligand trap. This agent induces durable RBC-TI, but there have been no reports of disease-modifying activity in terms of clone modifications, although there was a clinically meaningful and significant advantage in overall survival after 36 months of follow-up [9, 42–46]. Lenalidomide is also approved for the treatment of anemia in patients with RBC-TD MDS-del(5q) [5, 9, 47, 48]. Lenalidomide, an immunomodulatory thalidomide analog, has demonstrated responses in patients with LR-MDS [49], but its disease-modifying properties are exclusive for del(5q) LR-MDS, where del(5q) clones are specifically targeted for elimination. In some cases, del(5q) cells have been shown to persist due to resistance mechanisms [50], but recently, maintenance of long-term cytogenetic response at suspension of treatment has also been observed [51]. Hypomethylating agents (azacitidine and decitabine) have been shown to reduce the size of leukemic stem/progenitor cell compartments, but failed to provide complete eradication, even in patients who exhibited improvement of hematopoiesis or morphologic remission [52]. The oral combination of decitabine and cedazuridine offers a pharmacodynamically equivalent and more convenient alternative to the intravenous product, with similar efficacy and safety. Nevertheless, data are lacking regarding the effects on leukemic stem/progenitor cell populations for both the oral and parenteral options [53]. In contrast, the impact of the molecular modifications and cytogenetic responses presented here illustrate the possible disease-modifying properties of imetelstat, which are unique and evident in this LR-MDS patient population. The ability to induce molecular modifications is similar only to that observed for lenalidomide in MDS with del(5q) [48, 51]. Transient cytopenias induced by imetelstat treatment may be in line with this similarity in potential disease-modifying activity. Our observations suggest that not only is imetelstat a viable treatment option as monotherapy, but its mechanism of action and spectrum of activity may allow for potential long-lasting benefit in all WHO subtypes of LR-MDS. Combination with other active therapies should also be considered in the future.

Hematopoietic neoplasms/malignancies, including MDS, arise from hematopoietic stem cells that have been demonstrated to express higher TA and have shorter telomeres compared with healthy cells [54]. Patients with MDS and with high TA [26] or relatively shorter telomeres [55] have been shown to have a significantly shorter overall survival compared with those with lower TA or longer telomeres. Imetelstat targets TA and may thus selectively kill neoplastic HSPCs [13, 15, 56, 57]. Our data do not allow to determine whether the target cells of imetelstat are hematopoietic stem cells, progenitors, or more downstream populations. It seems from what we present here that imetelstat activity may enable polyclonal hematopoietic recovery, resulting in an increase in Hb and improvement of anemia. The results herein show that the response to imetelstat is accompanied by a reduction in TA/hTERT RNA levels. Due to the limited data in this analysis, it is not feasible to assess the correlation between baseline TA/hTERT RNA levels with ≥1-year RBC-TI response, although it was observed that 60% of ≥1-year RBC-TI responders had higher baseline hTERT RNA levels. This is of interest and further investigations on the mechanism of action of imetelstat in LR-MDS are ongoing. At present, the mechanism of action of imetelstat is not fully characterized, and it is unclear if its clinical efficacy is limited to telomerase inhibition via telomere shortening and/or via targeting other noncanonical functions of telomerase. It is of paramount relevance for this insight to inform investigations into which cell populations are the target cells for TA inhibition by imetelstat in MDS.

Consideration is needed when interpreting these findings beyond the standard drawbacks of retrospective analysis of prospectively collected clinical trial data. These data are descriptive in nature and obtained in a small sample size, thus are not adequately statistically powered to draw strong conclusions. However, it is important to highlight that specific properties of this agent were identified as a result of our focus on the biological modifications observed in the subgroup of patients with LR-MDS who had a significant improvement in Hb and long-lasting uninterrupted RBC-TI benefit with imetelstat treatment. In fact, the results presented provide a consistent base of evidence for disease-modifying properties of imetelstat that deserve further investigation. Additionally, longer follow-up is needed to determine if the potential disease-modifying activity observed with imetelstat translates into improved survival.

## Conclusions

In this exploratory analysis, treatment with imetelstat reduced or eliminated dysplastic clones in patients with RBC-TD non-del(5q) LR-MDS, particularly in patients who upon treatment achieved ≥1-year RBC-TI. The findings suggest that the first-in-class telomerase inhibitor imetelstat may modify the biology of LR-MDS.

## Supplementary information

Dataset 1

Supplement

## Data Availability

De-identified study data will be made available upon request to qualified researchers, to the extent permitted by applicable laws and participant informed consent. Approval of such requests is at the discretion of Geron Corporation and is dependent on the nature of the request, the merit of the research proposed, the availability of the data, and the intended use of the data. Data requests should be sent to medinfo@geron.com.
