# Supplementary material for: Modulation of the clonal burden in patients with lower-risk myelodysplastic neoplasms treated with imetelstat
Source: Leukemia. 2026 Jan 12;40(2):397–409. doi: 10.1038/s41375-025-02831-z (PMC12875879; doi:10.1038/s41375-025-02831-z)
Supplement: Supplementary file 1 — Dataset 1 [file 41375_2025_2831_MOESM1_ESM.pdf]

16.1.3 List of Ethics Committees

Site No.	Investigator Name	Name / Address of Ethics Committee
BE10001	Mazure, Dominiek	Ethische Commissie<br>UZ Gent<br>C Heymanslaan 10<br>900 Gent<br>Belgium
BE10002	Van Eygen, Koen	Ethische Commissie<br>UZ Gent<br>C Heymanslaan 10<br>900 Gent<br>Belgium
BE10004	Meers, Stef	Ethische Commissie<br>UZ Gent<br>C Heymanslaan 10<br>900 Gent<br>Belgium
BE10007	Selleslag, Dominik	Ethische Commissie<br>UZ Gent<br>C Heymanslaan 10<br>900 Gent<br>Belgium
BE10008	Heyrman, Bert	Ethische Commissie<br>UZ Gent<br>C Heymanslaan 10<br>900 Gent<br>Belgium
BE10050	Beckers, Marielle Maria	Ethische Commissie<br>UZ Gent<br>C Heymanslaan 10<br>900 Gent<br>Belgium
BE10051	Lemmens, Jan	Ethische Commissie<br>UZ Gent<br>C Heymanslaan 10<br>900 Gent<br>Belgium
CA10050	Zhu, Nancy	Health Research Ethics Board of Alberta<br>1500-Cancer Committee<br>1500,10104-103 Ave<br>Edmonton, Alberta<br>Canada T5J 0H8
CA10051	Buckstein, Rena	Ontario Cancer Research Ethics Board<br>661 University Avenue<br>MaRS Centre, Suite 510<br>Toronto, Ontario<br>Canada M5G 0A3

Site No.	Investigator Name	Name / Address of Ethics Committee
CA10052	Yee, Karen	Ontario Cancer Research Ethics Board<br>661 University Avenue<br>MaRS Centre, Suite 510<br>Toronto, Ontario<br>Canada M5G 0A3
CA10053	Geddes, Michelle	Health Research Ethics Board of Alberta<br>Cancer Committee<br>1500,10104-103 Ave<br>Edmonton, Alberta, Canada T5J 0H8
CA10054	Shamy, April	Medical/Biomedical (MBM) Research Ethics Committee of West Central Montreal Health<br>3755, Ch. De la Cote-Sainte-Catherine, Room A-925<br>Montreal, Quebec<br>H3T1E2
CA10055	Christou, Marie Grace	Ottawa Health Science Network Research Ethics Board (OHSN-REB)<br>725 Parkdale Ave<br>LOEB Building,<br>Ottawa, ON<br>K1Y 4E9
CZ10050	Mayer, Jiri	Central Ethics Committee:<br>Eticka komise<br>Fakultni nemocnice Kralovske Vinohrady<br>Srobarova 1150/50<br>100 34 Praha 10<br>Czech Republic<br><br>Local Ethics Committee:<br>Eticka komise<br>Fakultni nemocnice Brno<br>Jihlavska 20<br>625 00 Brno<br>Czech Republic
CZ10051	Belohlavkova, Petra	Central Ethics Committee:<br>Eticka komise<br>Fakultni nemocnice Kralovske Vinohrady<br>Srobarova 1150/50<br>100 34 Praha 10<br>Czech Republic<br><br>Local Ethics Committee:<br>Eticka komise<br>Fakultni nemocnice Brno<br>Jihlavska 20<br>625 00 Brno<br>Czech Republic

Site No.	Investigator Name	Name / Address of Ethics Committee
CZ10052	Cerna, Olga	Central Ethics Committee:<br>Eticka komise<br>Fakultni nemocnice Kralovske Vinohrady<br>Srobarova 1150/50<br>100 34 Praha 10<br>Czech Republic<br><br>Local Ethics Committee:<br>Eticka komise<br>Fakultni nemocnice Brno<br>Jihlavska 20<br>625 00 Brno<br>Czech Republic
CZ10053	Jonasova, Anna	Central Ethics Committee:<br>Eticka komise<br>Fakultni nemocnice Kralovske Vinohrady<br>Srobarova 1150/50<br>100 34 Praha 10<br>Czech Republic<br><br>Local Ethics Committee:<br>Eticka komise<br>Fakultni nemocnice Brno<br>Jihlavska 20<br>625 00 Brno<br>Czech Republic
FR10001	Fenaux, Pierre	Comite de Protectin des Personnes<br>Ile-de-France IV<br>Hopital Saint-Louis<br>Porte 5 du Carre Historique<br>ler etage - Centre de Fromation<br>1, avenue Claude-Vellefaux<br>75475 Paris Cedex 10/France
FR10002	Gourin, Marie-Pierre	Comite de Protectin des Personnes<br>Ile-de-France IV<br>Hopital Saint-Louis<br>Porte 5 du Carre Historique<br>ler etage - Centre de Fromation<br>1, avenue Claude-Vellefaux<br>75475 Paris Cedex 10/France
FR10004	Cluzeau, Thomas	Comite de Protectin des Personnes<br>Ile-de-France IV<br>Hopital Saint-Louis<br>Porte 5 du Carre Historique<br>ler etage - Centre de Fromation<br>1, avenue Claude-Vellefaux<br>75475 Paris Cedex 10/France

Site No.	Investigator Name	Name / Address of Ethics Committee
FR10005	Gyan, Emmanuel	Comite de Protectin des Personnes<br>Ile-de-France IV<br>Hopital Saint-Louis<br>Porte 5 du Carre Historique<br>ler etage - Centre de Fromation<br>1, avenue Claude-Vellefaux<br>75475 Paris Cedex 10/France
FR10006	Thepot, Sylvain	Comite de Protectin des Personnes<br>Ile-de-France IV<br>Hopital Saint-Louis<br>Porte 5 du Carre Historique<br>ler etage - Centre de Fromation<br>1, avenue Claude-Vellefaux<br>75475 Paris Cedex 10/France
FR10050	Park, Sophie	Comite de Protectin des Personnes<br>Ile-de-France IV<br>Hopital Saint-Louis<br>Porte 5 du Carre Historique<br>ler etage - Centre de Fromation<br>1, avenue Claude-Vellefaux<br>75475 Paris Cedex 10/France
FR10051	Original PI – Quesnel, Bruno<br>Subsequent PI – Goursaud, Laure	Comite de Protectin des Personnes<br>Ile-de-France IV<br>Hopital Saint-Louis<br>Porte 5 du Carre Historique<br>ler etage - Centre de Fromation<br>1, avenue Claude-Vellefaux<br>75475 Paris Cedex 10/France
FR10052	Torregrosa-Diaz, Jose Miguel	Comite de Protectin des Personnes<br>Ile-de-France IV<br>Hopital Saint-Louis<br>Porte 5 du Carre Historique<br>ler etage - Centre de Fromation<br>1, avenue Claude-Vellefaux<br>75475 Paris Cedex 10/France
FR10053	Guerci-Bresler, Agnes	Comite de Protectin des Personnes<br>Ile-de-France IV<br>Hopital Saint-Louis<br>Porte 5 du Carre Historique<br>ler etage - Centre de Fromation<br>1, avenue Claude-Vellefaux<br>75475 Paris Cedex 10/France
FR10054	Laribi, Kamel	Comite de Protectin des Personnes<br>Ile-de-France IV<br>Hopital Saint-Louis<br>Porte 5 du Carre Historique<br>ler etage - Centre de Fromation<br>1, avenue Claude-Vellefaux<br>75475 Paris Cedex 10/France

Site No.	Investigator Name	Name / Address of Ethics Committee
DE10001	Germing, Ulrich	Ethics Committee at the Medical Faculty<br>University of Düsseldorf<br>Moorenstraße 5<br>40225 Düsseldorf<br>Germany
DE10003	Sockel, Katja	Ethics Committee at the Medical Faculty<br>University of Düsseldorf<br>Moorenstraße 5<br>40225 Düsseldorf<br>Germany
DE10005	Lübbert, Michael	Ethics Committee at the Medical Faculty<br>University of Düsseldorf<br>Moorenstraße 5<br>40225 Düsseldorf<br>Germany
DE10006	Klausmann, Martine	Ethics Committee at the Medical Faculty<br>University of Düsseldorf<br>Moorenstraße 5<br>40225 Düsseldorf<br>Germany
DE10050	Platzbecker, Uwe	Ethics Committee at the Medical Faculty<br>University of Düsseldorf<br>Moorenstraße 5<br>40225 Düsseldorf<br>Germany
DE10051	Radsak, Markus	Ethics Committee at the Medical Faculty<br>University of Düsseldorf<br>Moorenstraße 5<br>40225 Düsseldorf<br>Germany
DE10052	Teichmann, Lino	Ethics Committee at the Medical Faculty<br>University of Düsseldorf<br>Moorenstraße 5<br>40225 Düsseldorf<br>Germany
DE10053	Illmer, Thomas	Ethics Committee at the Medical Faculty<br>University of Düsseldorf<br>Moorenstraße 5<br>40225 Düsseldorf<br>Germany
IL10050	Preis, Meir	The Helsinki Committee of the Lady David Carmel Medical Center<br>The Lady Davis Carmel Medical Center<br>7 Michal St<br>Haifa 3436212<br>Israel

Site No.	Investigator Name	Name / Address of Ethics Committee
IL10051	Winder, Asher	The Helsinki Committee of the E. Wolfson Medical Center<br>The E. Wolfson Medical Center<br>62 Halohamim St.<br>5810001<br>Israel
IL10052	Hellman, Ilana Nili	Helsinki Committee of Meir Medical Center<br>Meir Medical Center<br>59 Tshernichovsky St.,<br>Kfar-Saba 4428164<br>Israel
IL10053	Merkel, Drorit	Helsinki Committee of The Chaim Sheba Medical Center<br>The Chaim Sheba Medical Center<br>2, Sheba Road<br>Tel-Hashomer 5265601<br>Israel
IL10054	Wolach, Ofir	Helsinki Committee of Rabin Medical Center<br>39 Jabotinski St.<br>Petah Tikva 4941492<br>Israel
IL10055	Filanovsky, Kalman	Helsinki Committee of Kaplan Medical Center<br>Kaplan Medical Center<br>Hagalil St.<br>Rehovot 7610001<br>Israel
IL10056	Original PI – Bartfeld-Stemer, Galia<br>Subsequent PI - Chap-Marshak, Dafna<br>Subsequent PI – Okasha, Doaa	Helsinki Committee (IRB) of Ha'Emek Medical Center<br>Ha'Emek Medical Center<br>Yitshak Rabin Boulevard 21<br>Afula, 1834111<br>Israel
IL10057	Mittelman, Moshe	Helsinki Committee (IRB) of Tel Aviv Sourasky Medical Center<br>Tel Aviv Sourasky Medical Center<br>6 Weitzman St<br>Tel Aviv 6423906<br>Israel
IL10058	Lavie, David	Helsinki Committee (IRB) of Hadassah Medical Organization<br>Hadassah Medical Organization,<br>Hadassh Medical Center, Ein-Karem,<br>Kiryat Hadassah, PO Box 12000<br>Jerusalem 9112001<br>Israel

Site No.	Investigator Name	Name / Address of Ethics Committee
IT10002	Santini, Valeria	Comitato Etico Area Vasta Centro (CEA VC)<br>Azienda Ospedaliero - Universitaria Careggi<br>Largo Brambilla 3<br>50134 Firenze<br>Italy
IT10003	Tafuri, Agostino	Comitato Etico dell'Universita Sapienza<br>AOU Policlinico Umberto I<br>c/o Ufficio Locale Sperimentazioni Cliniche,<br>Azienda Ospedaliero Universitaria - Sant Andrea<br>Via di Gratarrossa 1035-1039<br>00189 Roma<br>Italy
IT10004	Oliva, Esther Natalie	Comitato Etico Regionale Sezione Area SUD0<br>Regione Calabria<br>c/o Grande Ospedale Metropolitano<br>Bianchi-Melacrino-Morelli<br>Via Provinciale Spirito Santo, 24<br>89128 Reggio Calabrina<br>Italy
IT10006	Voso, Maria Teresa	Comitato Etico Indipendente<br>Frondazione PTV - Policlinico Tor Vergata<br>Viale Oxford, 81<br>00133 Roma<br>Italy
IT10007	Cavo, Michele	Comitato Etico Indipendente<br>Area Vasta Emilia Centro (CE-AVEC)<br>Via Albertoni, 15<br>40138 Bologna, Italy
IT10008	Riva, Marta	Comitato Etico Milano Area 3<br>ASST Grande Ospedale Metropolitano Niguarda<br>Piazza Ospedale Maggiore, 3<br>20126 Milano<br>Italy
IT10050	Original PI: Musto, Pellegrino<br>Subsequent PI: Pietrantuono, Giuseppe	Comitato Etico Unico per la Basilicata (CEUR)<br>c/o Azienda Ospedaliera Regionale San Carlo<br>Via Potito Petrone, 1<br>8510 Potenza<br>Italy
IT10051	Poloni, Antonella	Comitato Etico Regione Marche<br>AOU Ospedali Riuniti<br>Umberto I - G.M. Lancisi - G. Salesi - Ospedale<br>Umberto 1<br>Via Conca, 71<br>60123 Torrette Ancona<br>Italy

Site No.	Investigator Name	Name / Address of Ethics Committee
IT10052	Original PI: Passamonti, Francesco<br>Subsequent PI: Barraco, Daniela	Comitato Etico dell'Insubria<br>c/o ASST Sette Laghi,<br>Viale Borri, 57<br>21100 Varese<br>Italy
IT10053	Della Porta, Matteo Giovanni	Comitato Etico<br>Instituto Clinico Humanitas<br>Via Manzoni, 56<br>20089 Rozzano (MI) - Italy
KR10001	Kim, Yoo-Jin	Institutional Review Board of<br>The Catholic University of Korea, Seoul<br>St. Mary's Hospital<br>222, Banpo-daero, Seocho-Gu<br>Seoul 06591<br>Korea
KR10002	Jang, Jun Ho	Institutional Review Board of Samsung<br>Medical Center<br>81, Irwon-ro, Gangnam-gu<br>Seoul, 06351<br>Republic of Korea
KR10003	Kim, Inho	Institutional Review Board of Seoul National<br>University Hospital<br>101 Daehak-Ro Jongno-Gu<br>Seoul, 03080<br>Republic of Korea
KR10004	Lee, Je-Hwan	Institutional Review Board of Asan Medical Center<br>88, Olympic-ro 43-gil, Songpa-gu<br>Seoul, 05505<br>Republic of Korea
KR10050	Cheong, June-won	Institutional Review Board of Severance Hospital,<br>Yonsei University Health System<br>50-1, Yonsei-ro, Seodaemun-gu<br>Seoul, 03722<br>Republic of Korea
KR10051	Shin, HoJin	Institutional Review Board of Pusan National<br>University Hospital<br>187, Gudeok-ro, Seo-gu<br>Busan, 49241<br>Republic of Korea
KR10052	Ahn, Jae-Sook	Institutional Review Board of Chonnam National<br>University Hwasun Hospital<br>322, Seoyang-ro, Hwasun-eup, Hwasun-gun<br>Jeollanam-do 58128<br>Korea

Site No.	Investigator Name	Name / Address of Ethics Committee
KR10053	Kim, Hawk	Institutional Review Board of Gachon University<br>Gil Medical Center<br>21 Namdong-daero 774 beon-gil, Namdong-gu<br>Incheon 21565<br>Republic of Korea
NL10001	van de Loosdrecht, Arjan A.	METc Universitair Medical Centrum<br>Groningen (UMCG)<br>Hanzeplein 1<br>9713 GZ Groningen<br>The Netherlands
NL10002	Langemeijer, Saskia M.C.	METc Universitair Medical Centrum<br>Groningen (UMCG)<br>Hanzeplein 1<br>9713 GZ Groningen<br>The Netherlands
NL10005	Original PI – Klein, Saskia K.<br>Subsequent PI – Fijnheer, Rob	METc Universitair Medical Centrum<br>Groningen (UMCG)<br>Hanzeplein 1<br>9713 GZ Groningen<br>The Netherlands
PL10050	Homenda, Wojciech	Komisja Bioetyczna<br>przy Okregowej Izbie Lekarskiej w Gdansku<br>Sniadeckich 33<br>80-204 Gdansk<br>Poland
PL10051	Halka, Janusz	Komisja Bioetyczna<br>przy Okregowej Izbie Lekarskiej w Gdansku<br>Sniadeckich 33<br>80-204 Gdansk<br>Poland
PL10052	Kazmierczak, Maciej	Komisja Bioetyczna<br>przy Okregowej Izbie Lekarskiej w Gdansku<br>Sniadeckich 33<br>80-204 Gdansk<br>Poland
PL10053	Wiszniewski, Pawel	Komisja Bioetyczna<br>przy Okregowej Izbie Lekarskiej w Gdansku<br>Sniadeckich 33<br>80-204 Gdansk<br>Poland
PL10055	Wrobel, Tomasz	Komisja Bioetyczna<br>przy Okregowej Izbie Lekarskiej w Gdansku<br>Sniadeckich 33<br>80-204 Gdansk<br>Poland

Site No.	Investigator Name	Name / Address of Ethics Committee
RU10001	Samoilova, Olga	Independent Interdisciplinary Ethics Committee on Ethical Review for Clinical Studies<br>51, Leningradskiy pr-t.<br>Moscow, 125468<br>Russia
RU10003	Original PI: Gritsaev, Sergey<br>Subsequent PI: Voloshin, Sergey	Local Ethics Committee at Federal State Budgetary Institution, Russian Research Institute of Haematology and Transfusiology of the Federal Biomedical Agency<br>16, 2-ya Sovetskaya str<br>Saint Petersburg, 191024<br>Russia
RU10006	Samarina, Irina	Independent Interdisciplinary Ethics Committee on Ethical Review for Clinical Studies<br>51, Leningradskiy pr-t.<br>Moscow, 125468<br>Russia
RU10009	Original PI – Udovitsa, Dmitry<br>Subsequent PI – Kirtbaya, Dmitry	Independent Interdisciplinary Ethics Committee on Ethical Review for Clinical Studies<br>5 l, Leningradskiy pr-t., Moscow, 125468, Russia
RU10012	Original PI – Doronin, Vadim<br>Subsequent PI – Chernova, Natalia	Local Ethics Committee at State Budgetary Institution of the city of Moscow, City Clinical Hospital #40 of the Healthcare Department of the city of Moscow<br>7, Kasatkina str.<br>Moscow, 129301<br>Russia
RU10050	Davydkin, Igor	Bioethics Committee at Federal State Budgetary Educational Institution of Higher Education, Samara State Medical University of the Ministry of Healthcare of the Russia Federation<br>20, Gagarina str.<br>Samara, 443079<br>Russia
ES10002	Diez Campelo, Maria	CEIm Hospital Universitari Vall d'Hebron<br>Secretaria del Comite Etico de Investigacion Clinica<br>Institut Recerca HUVH<br>Edif'icio Materno Infantil, planta 13<br>Passeig Vall d'Hebron, 119-129<br>08035 Barcelona<br>Spain

Site No.	Investigator Name	Name / Address of Ethics Committee
ES10003	Valcarcel Ferreiras, David	CEIm Hospital Universitari Vall d'Hebron<br>Secretaria del Comite Etico de Investigacion Clinica<br>Institut Recerca HUVH<br>Edif'icio Materno Infantil, planta 13<br>Passeig Vall d'Hebron, 119-129<br>08035 Barcelona<br>Spain
ES10004	Xicoy, Blanca	CEIm Hospital Universitari Vall d'Hebron<br>Secretaria del Comite Etico de Investigacion Clinica<br>Institut Recerca HUVH<br>Edif'icio Materno Infantil, planta 13<br>Passeig Vall d'Hebron, 119-129<br>08035 Barcelona<br>Spain
ES10005	de Paz Arias, Raquel	CEIm Hospital Universitari Vall d'Hebron<br>Secretaria del Comite Etico de Investigacion Clinica<br>Institut Recerca HUVH<br>Edif'icio Materno Infantil, planta 13<br>Passeig Vall d'Hebron, 119-129<br>08035 Barcelona<br>Spain
ES10006	Font Lopez, Patricia	CEIm Hospital Universitari Vall d'Hebron<br>Secretaria del Comite Etico de Investigacion Clinica<br>Institut Recerca HUVH<br>Edif'icio Materno Infantil, planta 13<br>Passeig Vall d'Hebron, 119-129<br>08035 Barcelona<br>Spain
ES10050	Sanz Santillana, Guillermo	CEIm Hospital Universitari Vall d'Hebron<br>Secretaria del Comite Etico de Investigacion Clinica<br>Institut Recerca HUVH<br>Edif'icio Materno Infantil, planta 13<br>Passeig Vall d'Hebron, 119-129<br>08035 Barcelona<br>Spain
ES10051	Original PI – Capota Huelva, Francisco Javier<br>Subsequent PI – Marchante Cepillo, Inmaculada	CEIm Hospital Universitari Vall d'Hebron<br>Secretaria del Comite Etico de Investigacion Clinica<br>Institut Recerca HUVH<br>Edif'icio Materno Infantil, planta 13<br>Passeig Vall d'Hebron, 119-129<br>08035 Barcelona<br>Spain

Site No.	Investigator Name	Name / Address of Ethics Committee
ES10052	Sayas Lloris, Maria Jose	CEIm Hospital Universitari Vall d'Hebron<br>Secretaria del Comite Etico de Investigacion Clinica<br>Institut Recerca HUVH<br>Edif'icio Materno Infantil, planta 13<br>Passeig Vall d'Hebron, 119-129<br>08035 Barcelona<br>Spain
ES10053	Vahi Sanchez de Medina, Maria	CEIm Hospital Universitari Vall d'Hebron<br>Secretaria del Comite Etico de Investigacion Clinica<br>Institut Recerca HUVH<br>Edif'icio Materno Infantil, planta 13<br>Passeig Vall d'Hebron, 119-129<br>08035 Barcelona<br>Spain
ES10054	Vara Pampliega, Miriam	CEIm Hospital Universitari Vall d'Hebron<br>Secretaria del Comite Etico de Investigacion Clinica<br>Institut Recerca HUVH<br>Edif'icio Materno Infantil, planta 13<br>Passeig Vall d'Hebron, 119-129<br>08035 Barcelona<br>Spain
ES10055	Ojeda Gutierrez, Emilio	CEIm Hospital Universitari Vall d'Hebron<br>Secretaria del Comite Etico de Investigacion Clinica<br>Institut Recerca HUVH<br>Edif'icio Materno Infantil, planta 13<br>Passeig Vall d'Hebron, 119-129<br>08035 Barcelona<br>Spain
CH10050	Original PI – Baerlocher, Gabriela Maria<br>Subsequent PI – Daskalakis, Michael	Department for Health, Social Affairs and Integration<br>Cantonal Research Ethics Committee<br>Murtenstrasse 31<br>CH-3010 Bern<br>Switzerland
CH10051	Silzle, Tobias	Department for Health, Social Affairs and Integration<br>Cantonal Research Ethics Committee<br>Murtenstrasse 31<br>CH-3010 Bern<br>Switzerland

Site No.	Investigator Name	Name / Address of Ethics Committee
CH10052	Balabanov, Stefan	Department for Health, Social Affairs and Integration<br>Cantonal Research Ethics Committee<br>Murtenstrasse 31<br>CH-3010 Bern<br>Switzerland
CH10053	Stehle, Gregor	Department for Health, Social Affairs and Integration<br>Cantonal Research Ethics Committee<br>Murtenstrasse 31<br>CH-3010 Bern<br>Switzerland
TU10050	Saydam, Guray	Ankara University Medical Faculty Clinical Trials Ethic Committee<br>Ankara Universitesi Tip Fakültesi Dekanligi<br>Klinik Arastirmalar Etik Kurulu 06100<br>Sihhiye/Ankara<br>Turkey
TU10051	Original PI – Ilhan, Osman<br>Subsequent PI – Toprak, Selami Kocak	Ankara University Medical Faculty Clinical Trials Ethic Committee<br>Ankara Universitesi Tip Fakültesi Dekanligi<br>Klinik Arastirmalar Etik Kurulu 06100<br>Sihhiye/Ankara<br>Turkey
TU10052	Sahin, Berksoy	Ankara University Medical Faculty Clinical Trials Ethic Committee<br>Ankara Universitesi Tip Fakültesi Dekanligi<br>Klinik Arastirmalar Etik Kurulu 06100<br>Sihhiye/Ankara<br>Turkey
UA10050	Original PI – Pylypenko, Halyna<br>Subsequent PI – Nogaieva, Larysa	Lokalna etchna komisiia kimunalnoho nekomertsiinoho pidpryiemstva<br>Cherkaskyi oblasnyi onkolohichnyi dyspanser<br>Cherkaskoi oblasnio rady<br>vul Mendelieieva 7<br>Cherkasy, 18009<br>Ukraine
UA10051	Maslyak, Zvenyslava	Komisiia zpytan etyky (Komitet z bioetyky ta deontolohii) pry Derzhavnii ustanovi, Instytut patolohii<br>krovi ta transfuziinoi medytsyny NAMN Ukrainy<br>vul. Henerala ChuorvnkV. 45. Lviv. 79044.<br>Ukraine

Site No.	Investigator Name	Name / Address of Ethics Committee
UA10052	Usenko, Ganna	Komisiia z pytan Miska klinichna likarnia No 4 Dniprovskoi miskoi rady miskyi hematolohichnyi tsentr vul. Blyzhnia 31 Dnipro, 49102 Ukraineetyky KNP (Komitet z bioetyky ta deontolohii)
GB10001	Culligan, Dominic	London- Brighton & Sussex Research Ethics Committee Health Research Authority Ground Floor, Skipton House 80 London Road, London, SE1 6LH United Kingdom
GB10006	Jain, Manish	London- Brighton & Sussex Research Ethics Committee Health Research Authority Ground Floor, Skipton House 80 London Road, London, SE1 6LH United Kingdom
GB10050	Original PI – Radia, Rohini<br>Subsequent PI – Byrne, Jennifer	London- Brighton & Sussex Research Ethics Committee Health Research Authority Ground Floor, Skipton House 80 London Road, London, SE1 6LH United Kingdom
GB10052	Narayanan, Srinivasan	London- Brighton & Sussex Research Ethics Committee Health Research Authority Ground Floor, Skipton House 80 London Road, London, SE1 6LH United Kingdom
US10002	Silverman, Lewis	Biomedical Research Alliance of New York (BRANY) 1981 Marcus Avenue, Suei 210 Lake Success, NY 11042
US10004	Jacoby, Meagan	Washington University School of Medicine Human Research Protection Office (HRPO) 660 South Euclid Ave, #8089 St Louis, MO 63110
US10007	Desai, Pinkal	Weill Cornell Medical College-Institutional Review Board 1300 York Ave, Box 89 New York, NY 10065
US10009	Original PI – Patel, Prapti<br>Subsequent PI – Madanat, Yazan	UT Southwestern Medical Center 5323 Harry Hines Boulevard BL9.100 Dallas, TX 75390

Site No.	Investigator Name	Name / Address of Ethics Committee
US10010	Schiller, Gary	UCLA Office of the Human Research Protection Program (OHRPP)<br>11000 Kinross Avenue<br>Suite 211<br>Los Angeles, CA 90095
US10012	Miller, Carole	Sterling Institutional Review Board<br>6300 Powers Ferry Road, Suite 600-351<br>Atlanta, GA 30339
US10014	Boccia, Ralph	Sterling Institutional Review Board<br>6300 Powers Ferry Road, Suite 600-351<br>Atlanta, GA 30339
US10017	Original PI – Sekeres, Mikkael<br>Subsequent PI – Patel, Bhumika<br>Subsequent PI – Carraway, Hetty	Cleveland Clinic Institutional Review Board<br>Cleveland Clinic Foundation<br>9500 Euclid Ave<br>Cleveland, OH 44195
US10021	Raza, Azra	Columbia University Medical Center - Institutional Review Board<br>1st Floor<br>New York, NY 10032
US10024	Original PI – Prebet, Thomas<br>Subsequent PI – Zeidan, Amer	Yale University IRB<br>#2, 3, 4B, 5, 6 – Human Investigation Committee<br>IB Oncology, II, III, IV, 1A<br>150 Munson St., 3rd Fl<br>New Haven, CT 06520
US10034	Scott, Bart	Fred Hutchinson Cancer Research Center<br>Institutional Review Office<br>1100 Fairview Ave, N<br>Seattle, WA 98109
US10050	Original PI – Arana Yi, Cecilia<br>Subsequent PI – Andritsos, Leslie	Western Institutional Review Board<br>1019 39th Ave SE, Suite 110<br>Puyallup, WA 98374
US10052	Original PI – Walker, Alison<br>Subsequent PI – Borate, Uma	Western Institutional Review Board<br>1019 39th Ave SE, Suite 110<br>Puyallup, WA 98374
US10054	Rustagi, Pradip Kumar	Western Institutional Review Board<br>1019 39th Ave SE, Suite 110<br>Puyallup, WA 98374
US10056	Amin, Bipinkumar	Western Institutional Review Board<br>1019 39th Ave SE, Suite 110<br>Puyallup, WA 98374
US10057	Agarwal, Ashwani	Sterling Institutional Review Board<br>6300 Powers Ferry Road, suite 600-351<br>Atlanta, GA 30339

Site No.	Investigator Name	Name / Address of Ethics Committee
US10059	Original PI – Azar, Catherine<br>Subsequent PI – Modiano, Manuel	Salus IRB<br>2111 West Braker Lane<br>Austin, TX 78758
US10060	Tache, Jason	Sterling Institutional Review Board<br>6300 Powers Ferry Road, suite 600-351<br>Atlanta, GA 30339
US10062	Komrokji, Rami	Advarra IRB<br>6940 Columbia Gateway Drive, Suite 110<br>Columbia, MD 21046
US10063	Mohan, Sanjay	Vanderbilt University Institutional Review Board<br>3319 West End Ave, suite 600<br>Nashville, TN 37203
