# Supplementary material for: Modulation of the clonal burden in patients with lower-risk myelodysplastic neoplasms treated with imetelstat
Source: Leukemia. 2026 Jan 12;40(2):397–409. doi: 10.1038/s41375-025-02831-z (PMC12875879; doi:10.1038/s41375-025-02831-z)
Supplement: Supplementary file 2 — Supplement [file 41375_2025_2831_MOESM2_ESM.docx]

**Supplementary Table 1.** ≥1-year RBC-TI rates in patients treated with imetelstat in subgroups of interest.

| **Categories, *n* (%)** | ***N*** | **≥1-year RBC-TI rate** |
| --- | --- | --- |
| **All patients**  95% CI | 118 | 21 (18)^a^  11-26^b^ |
| **Sex** | | |
| Female | 47 | 9 (19) |
| Male | 71 | 12 (17) |
| **Age (years)** | | |
| <65 | 27 | 5 (19) |
| ≥65 | 91 | 16 (18) |
| <75 | 83 | 14 (17) |
| ≥75 | 35 | 7 (20) |
| **Time since initial diagnosis** | | |
| <2 years | 31 | 6 (19) |
| ≥2 years | 87 | 15 (17) |
| **WHO classification** | | |
| RS+ | 73 | 15 (21) |
| RS− | 44 | 6 (14) |
| **Transfusion burden per IWG 2018** | | |
| HTB | 97 | 16 (16) |
| LTB | 21 | 5 (244) |
| **Prior ESA use** | | |
| Yes | 108 | 19 (18) |
| No | 10 | 2 (20) |
| **Baseline serum EPO levels** | | |
| ≤500 mU/mL | 87 | 18 (21) |
| >500 mU/mL | 26 | 3 (12) |
| **ECOG PS score** | | |
| 0 | 42 | 6 (14) |
| 1-2 | 76 | 15 (20) |
| **IPSS** |  |  |
| Low | 80 | 14 (18) |
| Intermediate-1 | 38 | 7 (18) |
| **IPSS-R (*n* = 111)** |  |  |
| Very low/low | 90 | 15 (17) |
| Intermediate/high | 21 | 4 (19) |
| **IPSS-M (*n* = 103)** |  |  |
| Very low/low/moderate low | 91 | 15 (16) |
| Moderate high/high/very high | 12 | 1 (8) |

*CI* confidence interval, *ECOG PS* Eastern Cooperative Oncology Group performance status, *EPO* erythropoietin, *ESA* erythropoiesis-stimulating agent, *HTB* high transfusion burden, *IPSS* International Prognostic Scoring System, *IPSS-M* Molecular International Prognostic Scoring System, *IPSS-R* Revised International Prognostic Scoring System, *IWG* International Working Group, *LTB* low transfusion burden, *RBC-TI* red blood cell transfusion independence, *WHO* World Health Organization.
^a^Percentage calculated from total population of 118 patients in Phase 3 of IMerge ^1^.
^b^The 95% CI is based on Wilson Score method.

**Supplementary Table 2.** Data summary of patients who achieved ≥1-year RBC-TI with imetelstat.

| **Patient ID** | **Time on treatment (months)** | **WHO 2016** | **IPSS** | **IPSS-R** | **IPSS-M** | **RBC transfusion units in 16 weeks before study entry** | **Baseline hTERT level** | **Longest transfusion-free interval (weeks)** | **Karyotype at baseline** | **Cytogenetic response** | **BM RS cell value at baseline** | **Central BM RS max absolute reduction** | **Central BM RS maximum % change** | **Mutant MDS genes at baseline*** | **Maximum % reduction in any mutation VAF** | **Maximum % reduction in TA/ hTERT** |
| --- | --- | --- | --- | --- | --- | --- | --- | --- | --- | --- | --- | --- | --- | --- | --- | --- |
| 1 | 37.82 | MDS-U | Low | Low | Low | 10 | >Median | 159.14 | Normal |  | Not applicable | Not applicable | Not applicable | *PTPN11, SF3B1, TET2* | −100 | −93.71 |
| 2 | 18.43 | RCMD-RS | Low | Low | Low | 11 | >Median | 144.86 | Normal |  | 94 | −94 | −100 | *DNMT3A, SF3B1* | −100 | −61.00 |
| 3 | 33.38 | RARS | Int-1 | Low | Very low | 10 | >Median | 141.86 | 45,X,-Y[19]/ 46,XY[1] | PR | 90 | −90 | −100 | *SF3B1* | −100 | −83.36 |
| 4 | 32.46 | RARS | Low | Low | Moderate high | 6 | >Median | 136.86 | Normal |  | 92 | −67 | −72.83 | *SF3B1, TET2* | −14.16 | −94.72 |
| 5 | 27.86 | MDS-U | Low | Low | Low | 9 | ≤Median | 132.14 | Normal |  | Not applicable | Not applicable | Not applicable | *SF3B1, TET2* | −100 | No reduction |
| 6 | 41.43 | RCUD-RA | Int-1 |  | N/A | 6 | >Median | 122.86 | N/A |  | Not applicable | Not applicable | Not applicable | *SETBP1, SF3B1* | −82.43 | −34.55 |
| 7 | 28.98 | RCMD-RS | Low | Int | Low | 12 | >Median | 121.00 | 47,XX,+8[3]/46, XX[17] | CR | 96 | −92 | −95.83 | *CUX1, JAK2, SF3B1, TET2* | −100 | −48.67 |
| 8 | 27.17 | RARS | Low | Low | Low | 10 | >Median | 118.00 | Normal |  | 86 | −86 | −100 | *DNMT3A, SF3B1, TET2* | −85.22 | −71.32 |
| 9 | 25.13 | RCUD-RA | Low | Low | Moderate low | 12 | ≤Median | 105.14 | 47,XY,+21[2]/46, XY[18] | CR | Not applicable | Not applicable | Not applicable | *ASXL1, SF3B1, TET2* | −100 | −54.96 |
| 10 | 23.20 | RCMD | Low | Low | Very low | 6 | ≤Median | 99.00 | Normal |  | Not applicable | Not applicable | Not applicable | *ATM, CUX1, SF3B1, TET2* | −100 | No reduction |
| 11 | 26.22 | RARS | Low | Low | Low | 12 | >Median | 93.86 | Normal |  | 80 | −73 | −91.25 | *JAK2, SF3B1, TET2* | −100 | −68.31 |
| 12 | 22.47 | RARS | Low | Low | Very low | 8 | ≤Median | 87.71 | Normal |  | 72 | −30 | −41.67 | *SF3B1, TET2* | −60.54 | −93.79 |
| 13 | 23.95 | RCMD-RS | Low | Low | Low | 12 | ≤Median | 83.86 | Normal |  | N/A | N/A | N/A | *SF3B1, TET2* | −28.41 | −47.47 |
| 14 | 21.29 | RARS | Int-1 | Low | Moderate low | 6 | >Median | 80.43 | Normal |  | 87 | −27 | −31.03 | *ASXL1, BCOR, DNMT3A, PTEN, SF3B1* | −54.31 | −52.05 |
| 15 | 22.34 | RARS | Int-1 | Int | Low | 8 | ≤Median | 80.00 | 46,XX,DEL(13) (Q12Q14) (12)/47,IDEM,+8[8] | PR | 88 | −36 | −40.91 | *SF3B1* | −100 | −70.03 |
| 16 | 17.77 | RCMD-RS | Int-1 | Int | Low | 10 | ≤Median | 63.86 | 45,X,-Y[4]/47,XY, +8[3]/46,XY[13] | CR | 94 | −84 | −89.36 | *SF3B1* | −84.95 | −62.18 |
| 17 | 24.44 | RARS | Int-1 | Low | Low | 14 | >Median | 62.00 | Normal |  | 96 | −93 | −96.88 | *SF3B1* | −65.38 | −47.56 |
| 18 | 17.28 | RCMD-RS | Low |  | N/A | 9 | >Median | 59.86 | N/A |  | 88 | −73 | −82.95 | *DNMT3A*, *SF3B1*, *TET2* | −100 | −43.49 |
| 19 | 43.50 | RCMD | Low | Low | N/A | 11 | ≤Median | 184.57 | 46,XY,DEL(13) (Q12Q14)[3]/ 46,XY[17] | CR | Not applicable | Not applicable | Not applicable | N/A | N/A | No reduction |
| 20 | 31.80 | RARS | Int-1 | Int | N/A | 7 | N/A | 159.71 | 47,XX,+8[5] | CR | 89 | −86 | −96.63 | N/A | N/A | N/A |
| 21 | 30.32 | RCMD-RS | Low | Low | N/A | 12 | >Median | 94.71 | Normal |  | 33 | −15 | −45.45 | N/A | N/A | −78.23 |

*Only baseline mutations with VAF ≥10% are included.

*BM*, bone marrow; *CR*, complete response; *hTERT*, human telomerase reverse transcriptase; *Int*, Intermediate; *IPSS*, International Prognostic Scoring System; *IPSS-M*, Molecular International Prognostic Scoring System; *IPSS-R*, Revised International Prognostic Scoring System; *MDS-U*, unclassifiable myelodysplastic syndromes; *N/A*, not available; *PR*, partial response; *RARS*, refractory anemia with ring sideroblasts; *RBC*, red blood cell; *RCMD*, refractory cytopenia with multilineage dysplasia; *RCUD-RA*, refractory cytopenia with unilineage dysplasia; *RS*, ring sideroblast; *TA*, telomerase activity; *TERT*, telomerase reverse transcriptase; *TI*, transfusion independence.

**Supplementary Table 3.** Baseline characteristics of the patients with sequential t-NGS and MFC data from bone marrow mononuclear cells (*n* = 4).

|  | **Mean ± SD** | **Median (min-max)** |
| --- | --- | --- |
| **Patients characteristics**  Age, y  M:F ratio | 75.2 ± 3.3  1:1 | 75 (72-79)  - |
| **Hematology**  Hb, g/dL  Platelet, n x10^9^/L  ANC, n x10^9^/L  BM blasts, %  sEPO (U/L)  sEPO <200 U/L, n (%) | 8.3 ± 0.84  287.8 ± 93.3  2.85 ± 0.54  2.1 ± 0.73  120.1 ±111.3  3 (75) | 8.8 (7-9)  287 (166-391)  3.08 (2-3)  0-3  92.9 (17-277)  - |
| **WHO 2022, n (%)**  MDS-LB  MDS-*SF3B1* | 1 (25)  3 (75) | -  - |
| **MDS prognosis**  IPSS-R score  IPSS-M score | 2.5 ± 0.41  -0.99 ± 0.61 | 2.5 (2-3)  -1.2 (-1.5- -0.2) |
| **Molecular features**  Mutations/patient, n | 2.5 | - |
| **Mutated genes**  *TET2*, n (%)  *SF3B1*, n (%)  *IDH2*, n (%)  Dominant gene VAF | 3 (75)  3 (75)  1 (25)  29.9 ± 6.6 | -  -  -  30.1 (22.4-37.1) |
| **Transfusion burden**  RBC U/8 weeks  HTB, n (%) | 7.8 ±2.6  4 (100) | -  8 (5-10) |
| **HI-E (IWG 2018)**  Response rate, n (%) | 2 (50) | - |

*ANC* absolute neutrophilic count, *BM* bone marrow, *Hb* Hemoglobin, *HI-E* erythroid hematologic improvement (as per IWG 2018 criteria); *HTB* high transfusion burden (as per IWG 2018 criteria), *IWG* International Working Group, *sEPO* serum erythropoietin, *MDS-LB* myelodysplastic syndromes with low blast count; *MFC* multiparametric flow cytometry, *RBC U* transfused red blood cell units, *SD*, standard deviation, *t-NGS* targeted next generation sequencing, *VAF*, variant allele fraction.

**Supplementary** **Fig. 1 VAF quantitation^a^ over time in individual patients who achieved ≥1-year RBC-TI with imetelstat.**


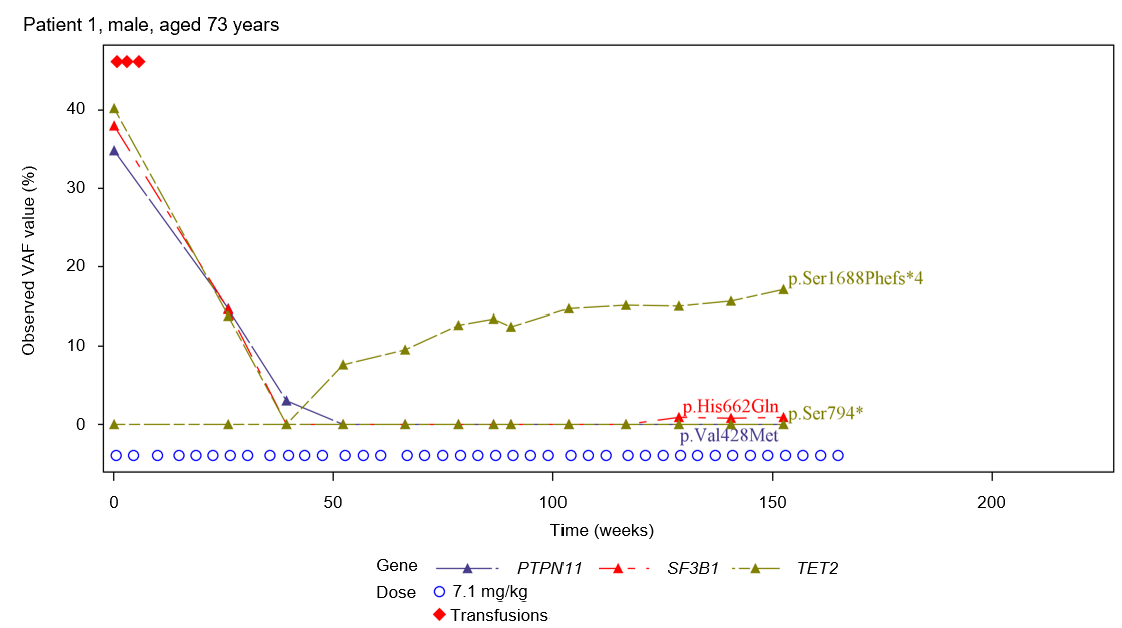


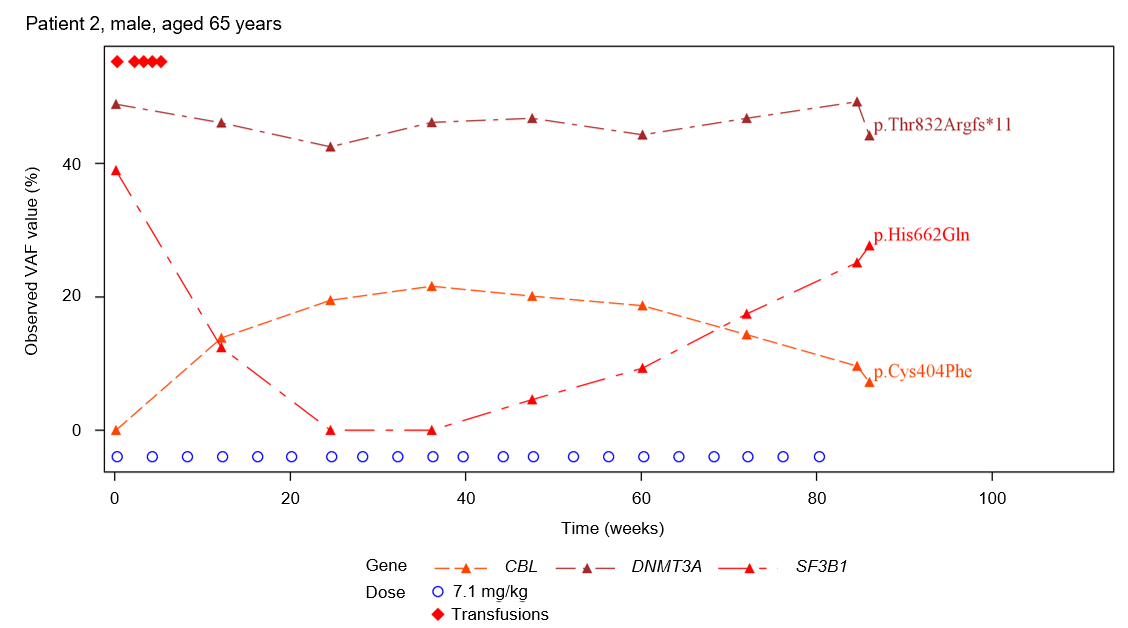


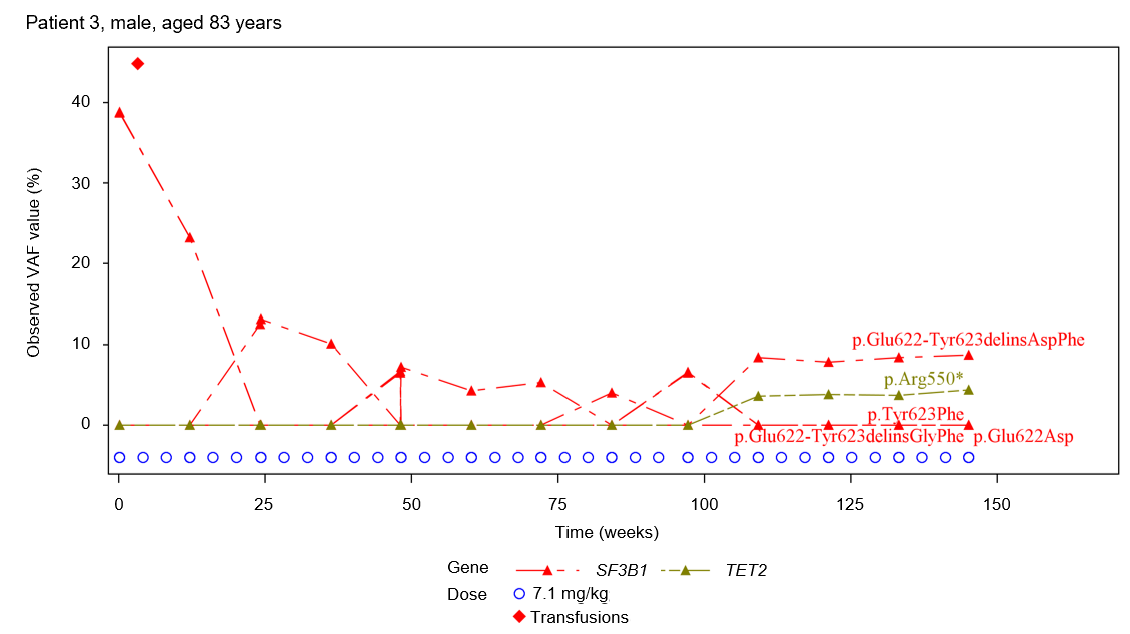


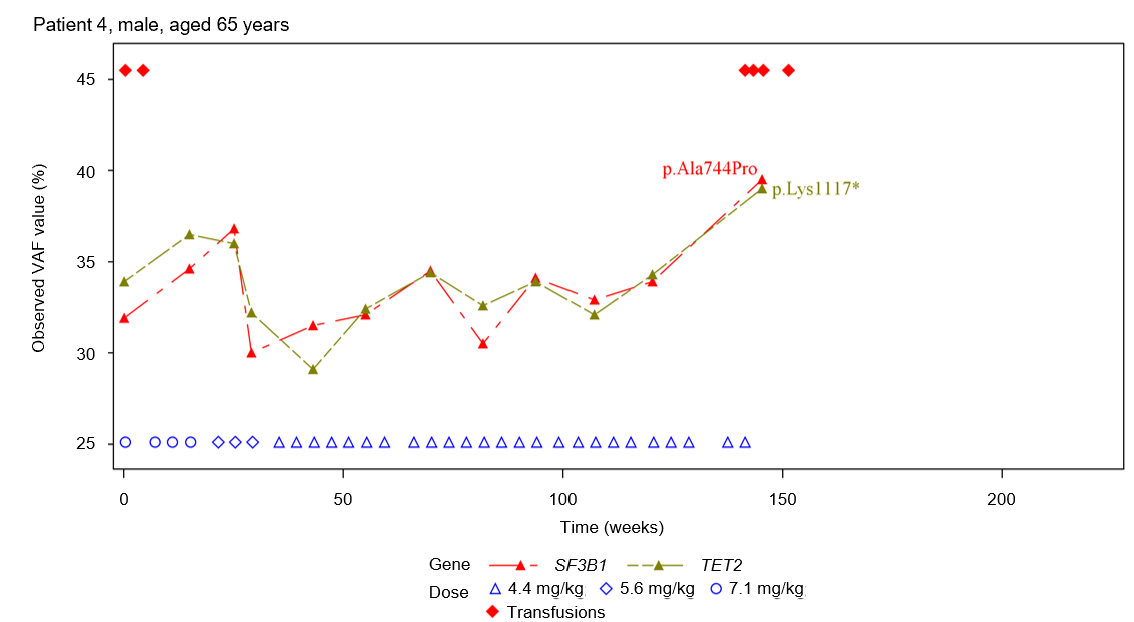


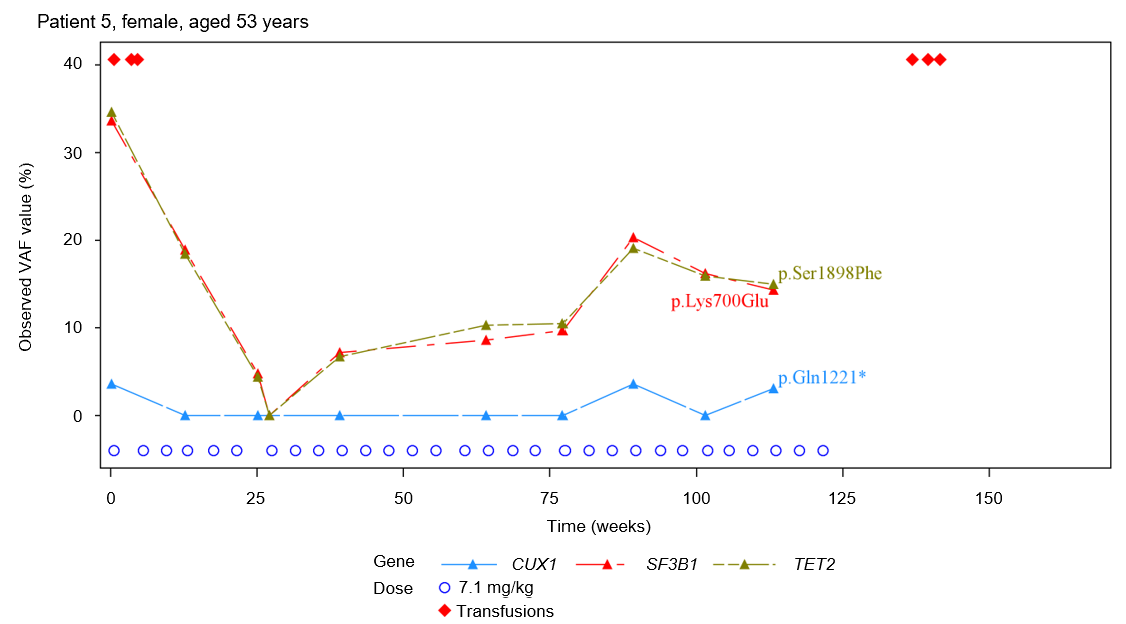


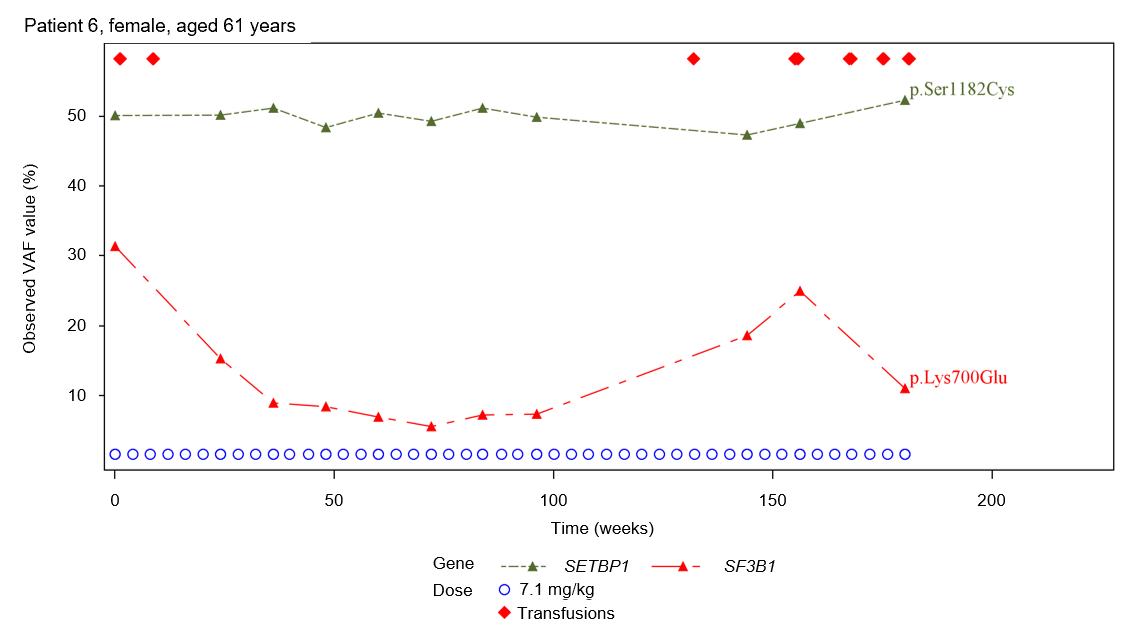


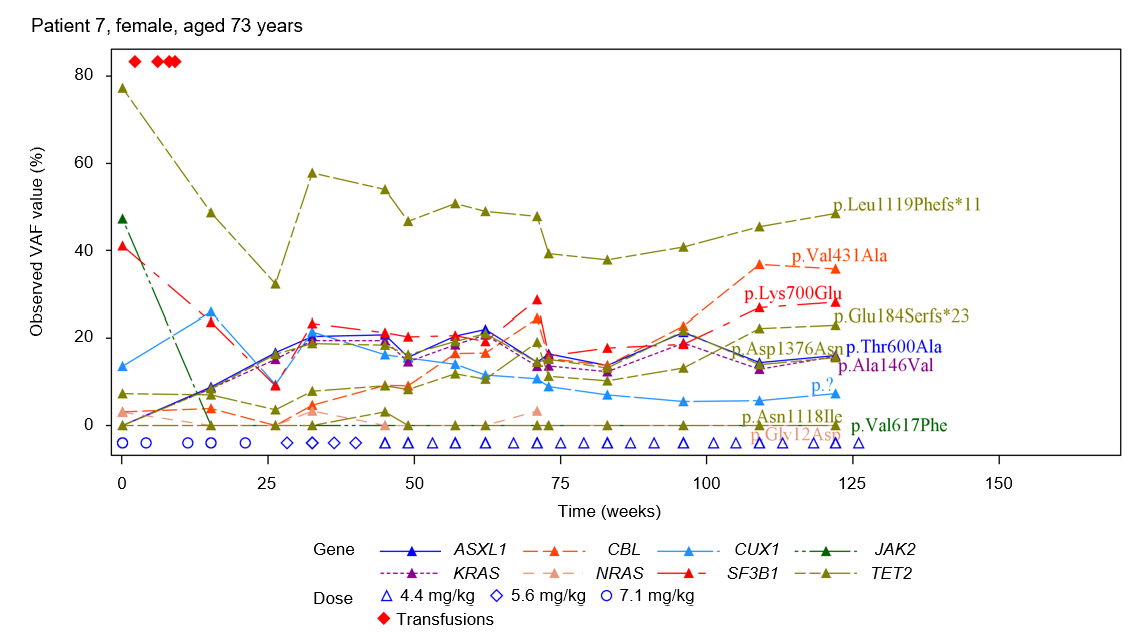


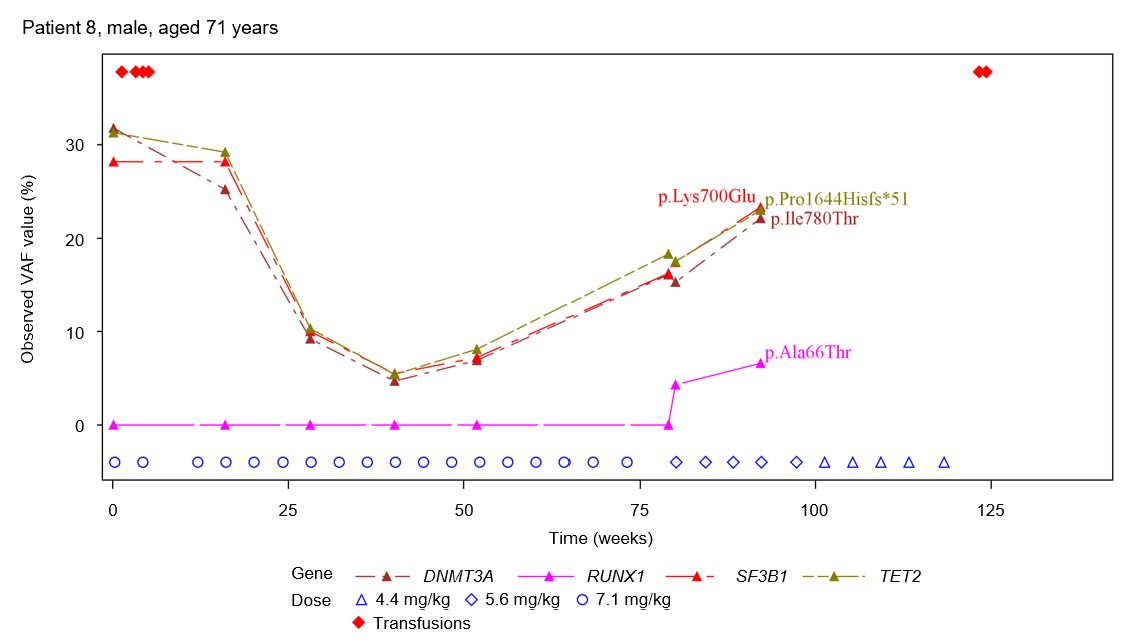


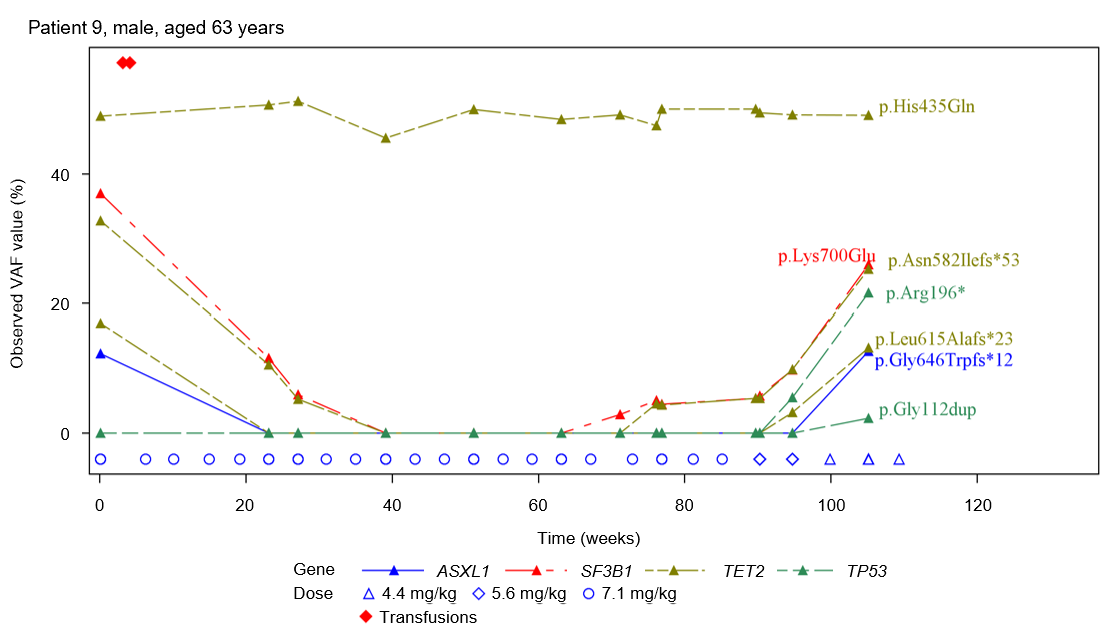


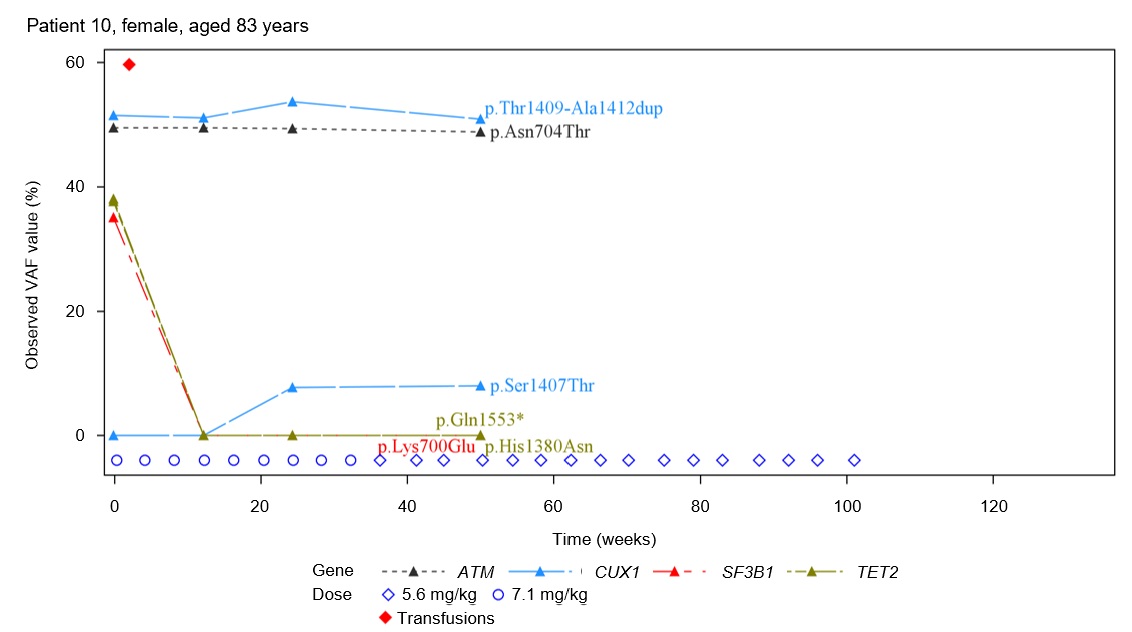


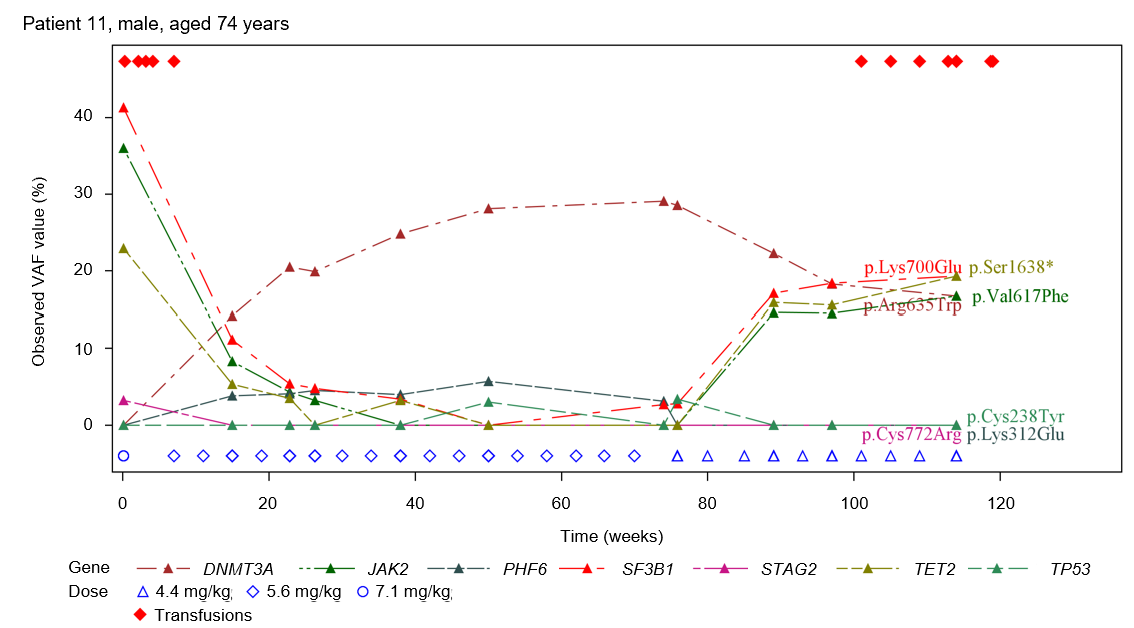


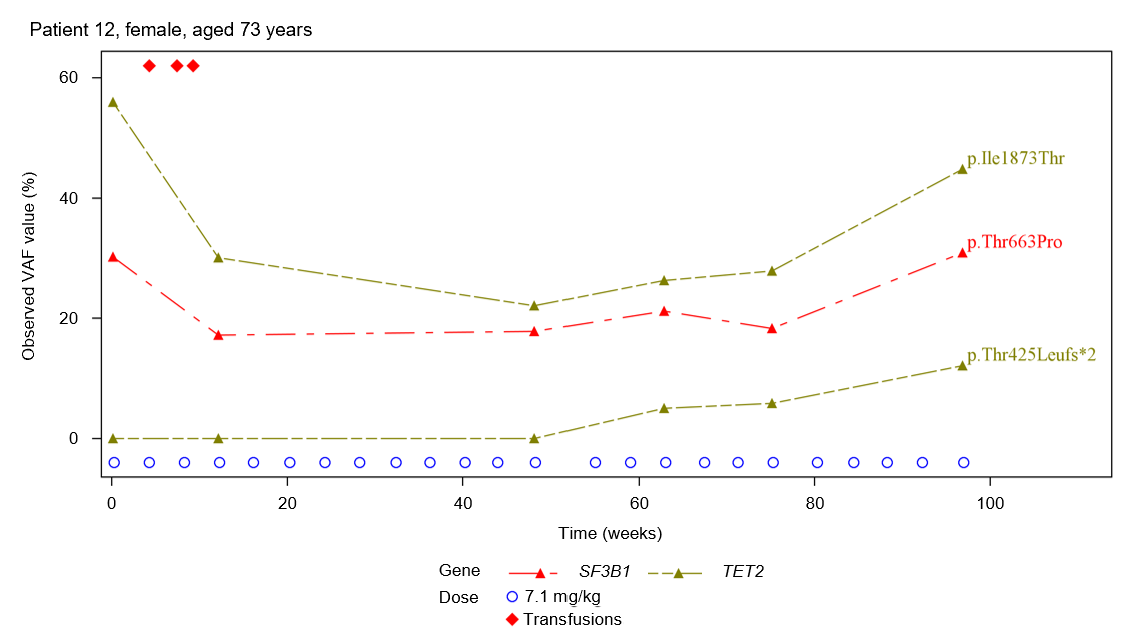


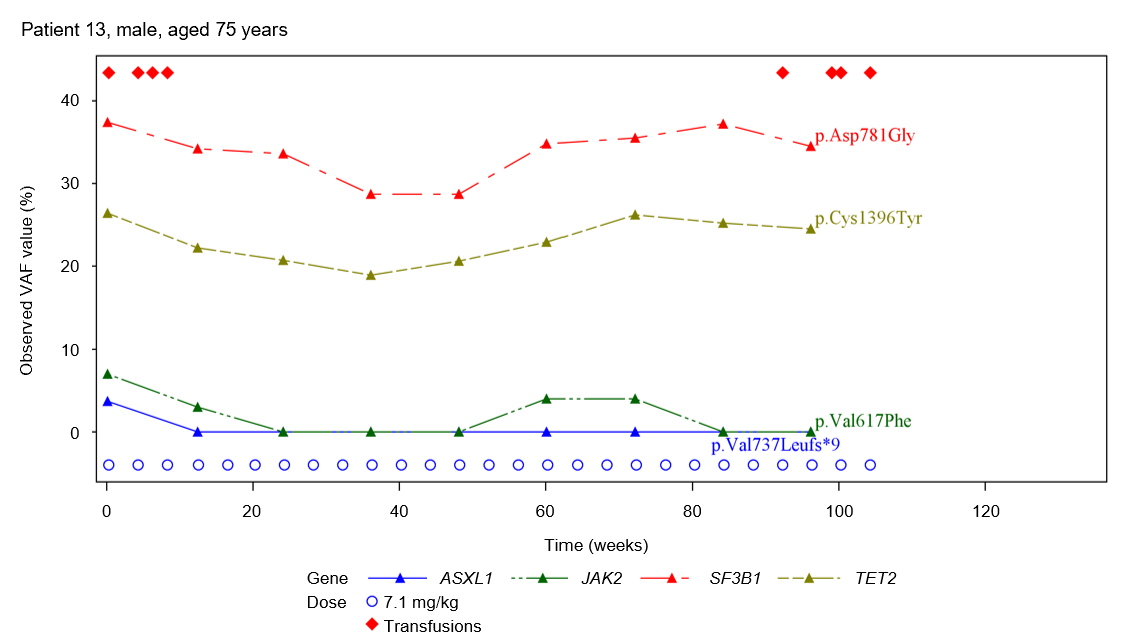


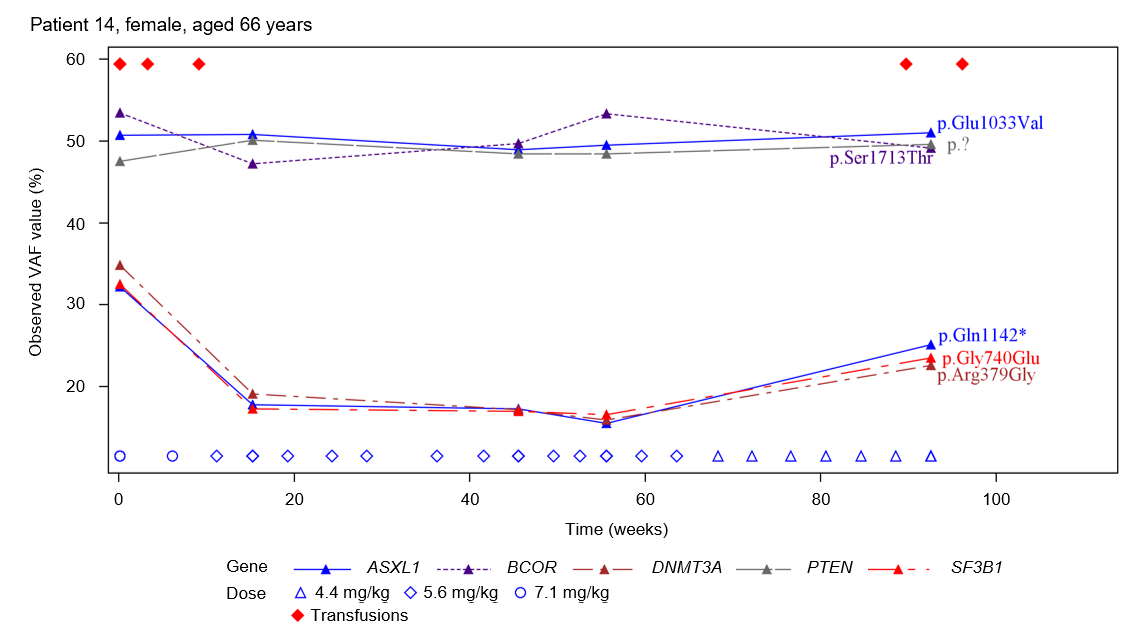


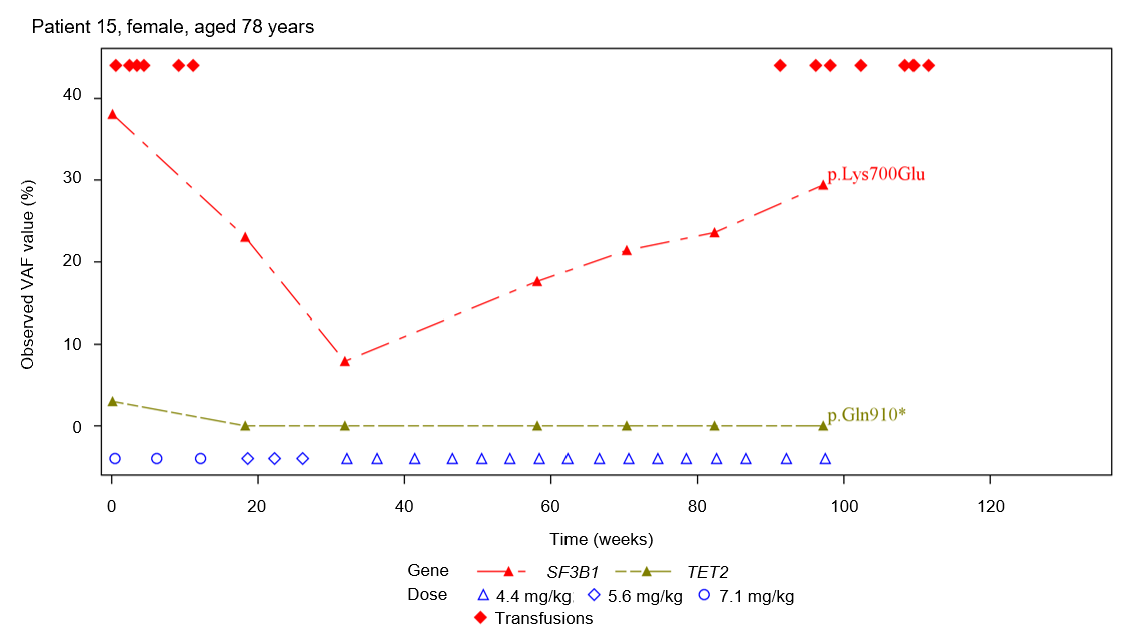


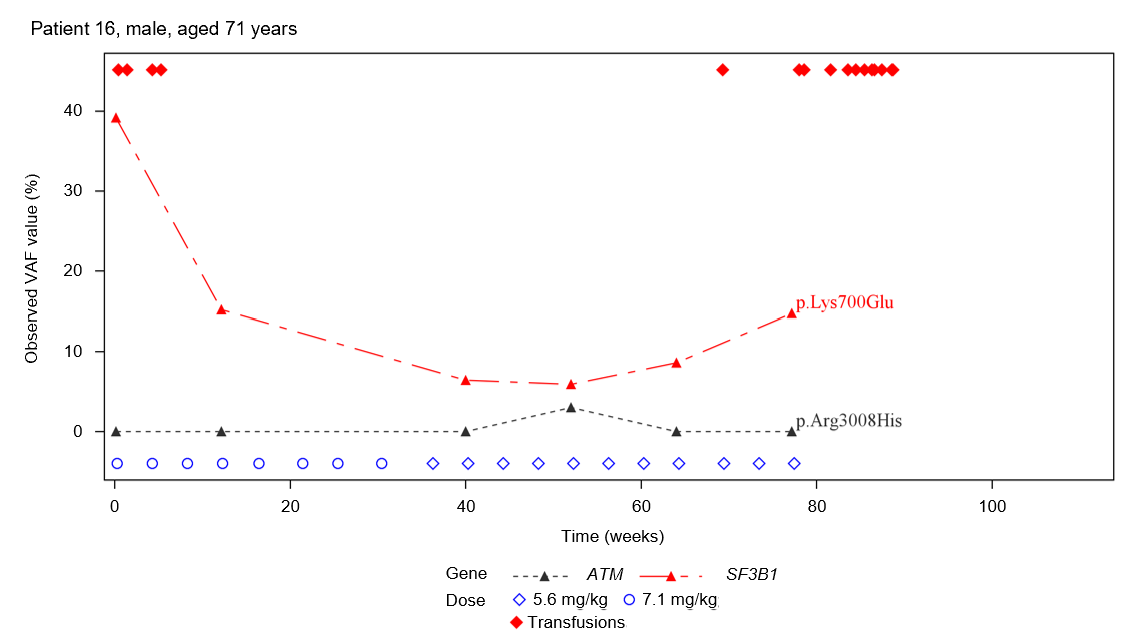


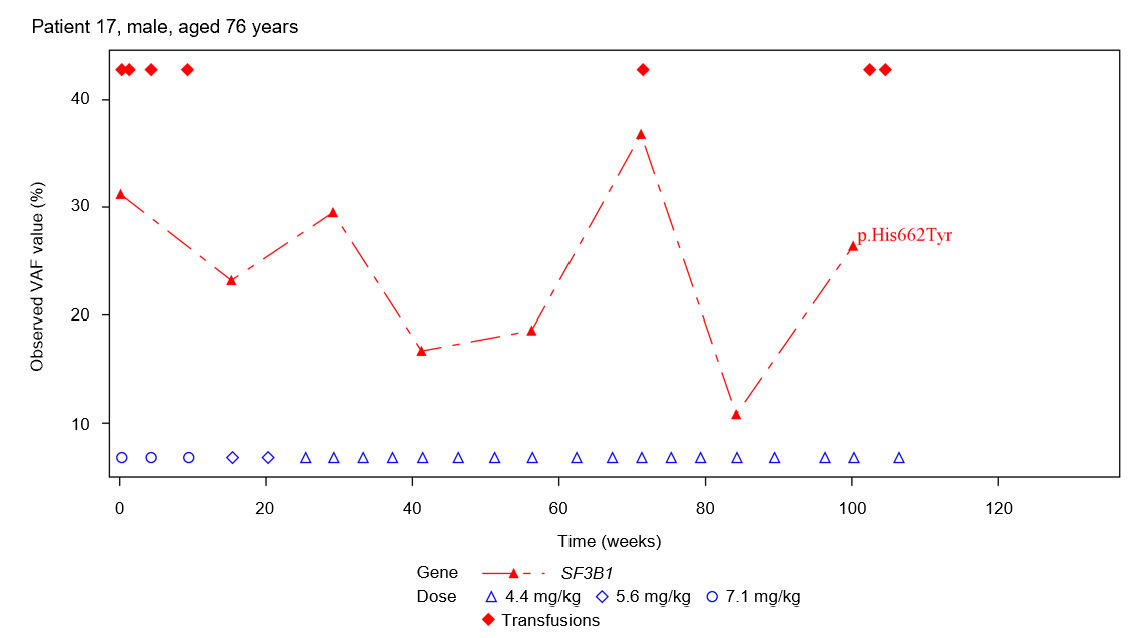


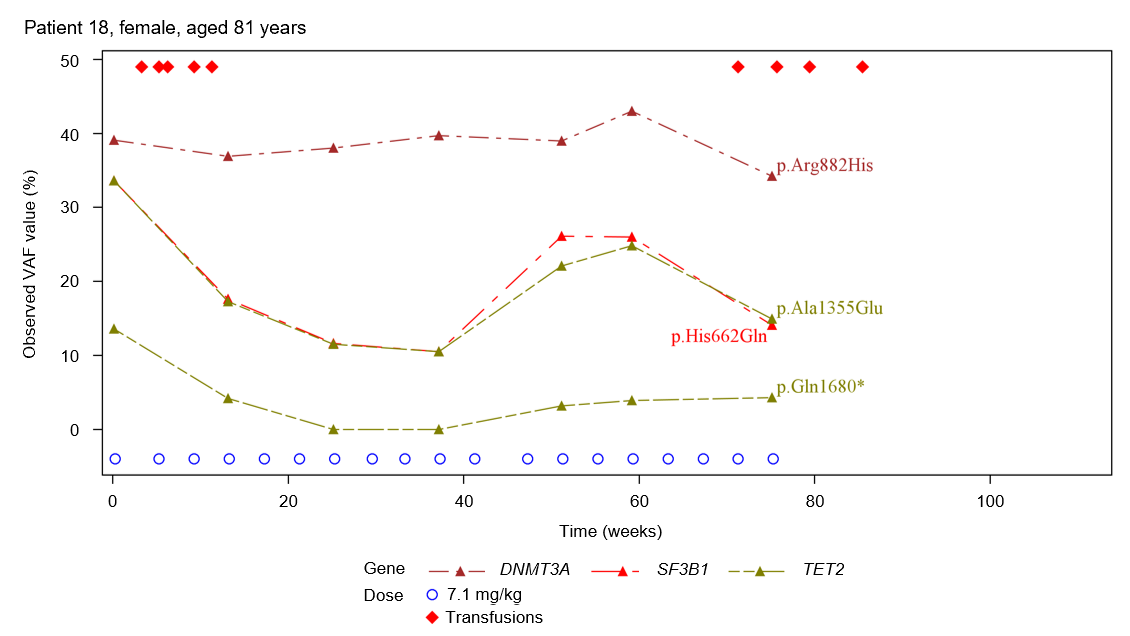


^a^All VAF values are presented as observed without any exclusion based on baseline VAF ≥10%. *RBC* red blood cell, *TI* transfusion independence, *VAF* variant allele frequency.

**Supplementary Fig. 2** **Predicted probability of achieving ≥1-year RBC-TI based on maximum VAF reduction from baseline in patients treated with imetelstat.**


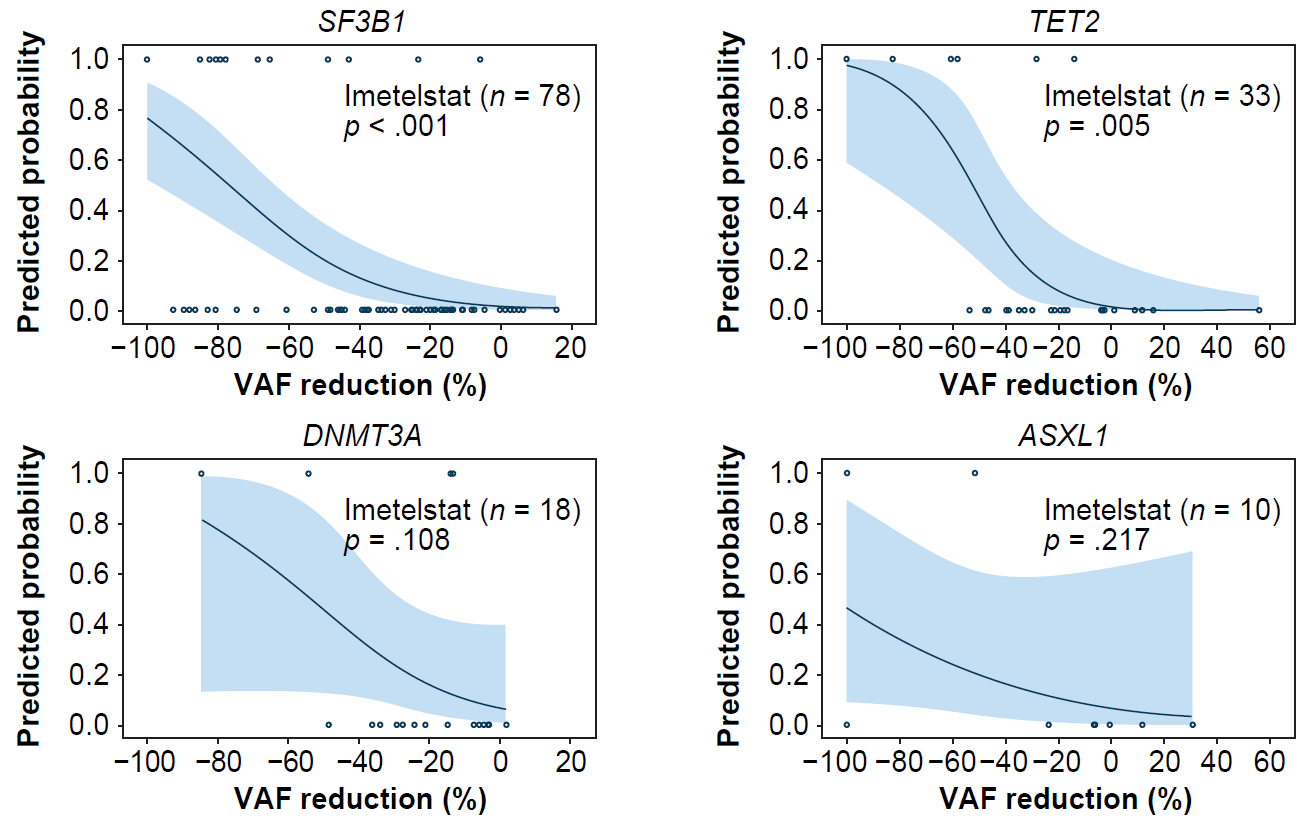


*RBC* red blood cell, *TI* transfusion independence, *VAF* variant allele frequency.

**Supplementary Fig. 3** **Changes in VAF and erythroid maturation during treatment with imetelstat/placebo in the patients with sequential t-NGS and MFC data from bone marrow mononuclear cells (*n* = 4).** Erythroid maturation indices (e.g., total erythroid cell and late/early erythroid precursors ratio) are also depicted and correlate with VAF changes.


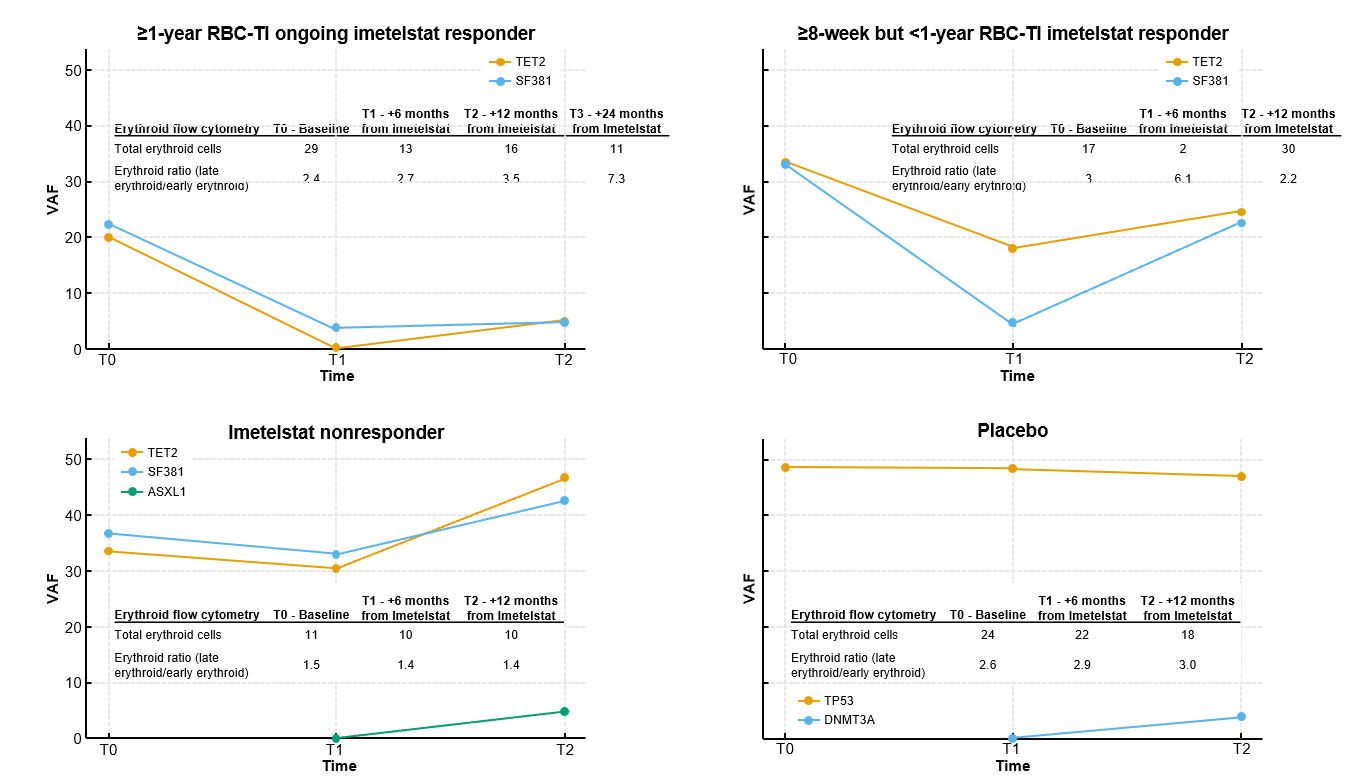


*MFC* multiparametric flow cytometry, *NGS* next-generation sequencing, *RBC* red blood cell, *TI* transfusion independence, *t-NGS* targeted next-generation sequencing, *VAF* variant allele frequency.

**References**

1. Platzbecker U, Santini V, Fenaux P, Sekeres MA, Savona MR, Madanat YF*, et al.* Imetelstat in patients with lower-risk myelodysplastic syndromes who have relapsed or are refractory to erythropoiesis-stimulating agents (IMerge): a multinational, randomised, double-blind, placebo-controlled, phase 3 trial. *Lancet* 2024 Jan 20; **403**(10423)**:** 249-260.
